# The Landscape of Long Non‐Coding RNAs Provides Insights Into the Domestication and Improvement of Pear Fruit

**DOI:** 10.1002/advs.77091

**Published:** 2026-08-19

**Authors:** Bobo Song, Peilin Han, Jiaming Li, Xiaolong Li, Xiang Zhang, Jing Fan, Dongliang Liu, Jianfa Cai, Lingchao Zhang, Jun Wu

**Affiliations:** ^1^ College of Horticulture State Key Laboratory of Crop Genetics & Germplasm Enhancement and Utilization Nanjing Agricultural University Nanjing Jiangsu China; ^2^ School of Horticulture Anhui Agricultural University Hefei Anhui China; ^3^ Key Laboratory of Quality and Safety Control for Subtropical Fruit and Vegetable Ministry of Agriculture and Rural Affairs College of Horticulture Science Zhejiang A&F University Hangzhou Zhejiang China; ^4^ College of Horticulture Science and Engineering Shandong Agricultural University Tai'an Shandong China; ^5^ Institute of Fruit and Tea Hubei Academy of Agricultural Sciences Wuhan China

**Keywords:** DNA methylation, domestication and improvement, long non‐coding RNA, metabolome, pear

## Abstract

Long non‐coding RNAs (lncRNAs) play essential roles in plant growth and development. However, their expression variability and contribution to phenotypic changes during the domestication of fruit crops remain unclear. Here, we generated approximately 3.5T of strand‐specific RNA sequencing data from wild, landrace, and improved pear varieties and identified 12 422 lncRNAs that exhibited higher tissue specificity and lower sequence conservation than protein‐coding genes. Roughly 74% of lncRNAs overlapped with transposable elements (TEs) and significantly impacted sequence diversity, expression variation, and tissue‐specific expression. DNA methylation explained approximately 32% of the expression variation of lncRNAs across accessions. Selective sweeps revealed 857 and 519 lncRNAs under domestication and improvement, respectively. These selected lncRNAs were associated with traits such as fruit size, sugar content, and stone cell content. Metabolome profiling revealed a consistent reduction in lignin‐related metabolites during pear domestication and improvement. Integrating transcriptome and metabolome analyses, we established a lncRNA‐mRNA co‐expression network associated with lignin biosynthesis. The regulatory role of *lncRNA‐pys* within this network was functionally validated through transgenic experiments, demonstrating that overexpressing *lncRNA‐pys* led to an increase in lignin content in pear. This study represents the first effort to unveil the contributions of lncRNAs under human selection to the domestication traits of fruit crops.

## Graphical abstract

This study reveals that long non‐coding RNAs represent important regulatory components underlying pear domestication and improvement. Integrative multi‐omics analyses identify selected lncRNAs associated with fruit traits, while functional validation uncovers a lignin‐related lncRNA, *lncRNA‐pys*, that promotes lignin accumulation.

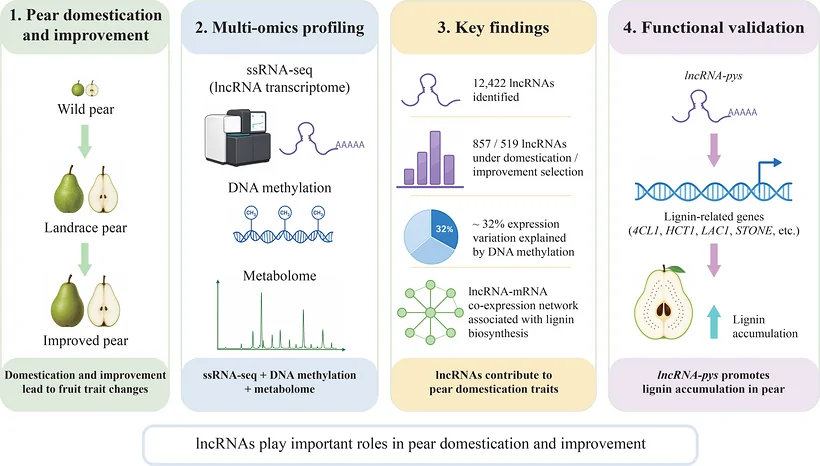

## 1 Introduction

In eukaryotes, approximately 75% of the genome can be transcribed, although merely 1%–2% of it can encode proteins [1, 2, 3]. This indicates that alongside a limited number of protein‐coding genes (PCGs), there are still a large number of non‐coding RNAs [4, 5]. Long non‐coding RNAs (lncRNAs) are emerging as a novel class of non‐coding RNAs, and a growing body of research has demonstrated their involvement in various critical biological processes, including floral transformation [6], pollen development [7], anthocyanin accumulation [8, 9, 10] and stress responses [11, 12]. According to their genomic positions relative to protein‐coding genes, plant lncRNAs are commonly classified into long intergenic non‐coding RNAs (lincRNAs), intronic non‐coding RNAs (incRNAs), and natural antisense transcripts (NATs). Different classes of lncRNAs may exert distinct regulatory effects because of their genomic organization. lincRNAs are generally considered to regulate neighboring or distal genes in cis or trans, incRNAs may be involved in the regulation of host gene transcription or RNA processing, and NATs may influence overlapping sense genes through antisense‐dependent regulation, including transcriptional interference, RNA stability modulation, alternative splicing, and epigenetic control [13, 14]. Thanks to the rapid advancement of high‐throughput sequencing technologies, the identification of genome‐wide lncRNAs has become feasible, and their roles have been extensively explored in numerous model plants [15, 16, 17, 18]. In *Arabidopsis*, more than 6000 lincRNAs have been identified using tiling arrays and transcriptome sequencing [19]. By leveraging sequence annotation and RNA‐seq datasets from 30 experiments, a total of 20 163 potential lncRNAs were identified in maize. Remarkably, over 90% of these lncRNAs served as precursors of miRNAs [20]. In rice, a total of 3363 high‐confidence lncRNAs were identified, including 2517 lincRNAs, 200 incRNAs, and 646 NATs. These findings were based on an analysis of 19 representative rice samples, encompassing 10 cultivated and 9 wild rice varieties [21]. Advances in high‐throughput sequencing technologies, particularly strand‐specific RNA sequencing (ssRNA‐seq), have made it possible to quantify lncRNA expression and explore their roles at the whole‐genome level.

The regulatory functions of plant lncRNAs have been characterized in several species. For example, in *Arabidopsis*, multiple lncRNAs are involved in the regulation of the floral repressor *FLC*. Among them, the antisense lncRNA *COOLAIR* contributes to cold‐induced transcriptional repression and chromatin‐state switching during vernalization [22], whereas the intronic lncRNA *COLDAIR* is required for PRC2‐mediated repression of *FLC* in the vernalization response [23]. In addition, the antisense lncRNA *ASL* has been implicated in *FLC* silencing in the autonomous flowering pathway [24]. In apple, the lncRNA *MdLNC499* enhances the expression of its neighboring gene *MdERF109*, an ethylene response factor that positively regulates anthocyanin biosynthesis under light conditions [9]. In chrysanthemum, the lncRNA *DglncTCP1* enhances the expression of *DgTCP1*, a gene that improves cold tolerance by reducing ROS accumulation [25]. In oolong tea, transcriptome analysis identified lncRNAs *LTCONS_00026271* and *LTCONS_00020084* as being enriched in flavonoid biosynthesis, terpenoid metabolism, and JA/MeJA signaling pathways [26]. In pear, Wang et al. identified two lncRNAs, *Ethylene Inhibiting Factor 1* (*EIF1*) and *EIF2*, which are involved in the regulation of fruit ripening by modulating both ethylene biosynthetic genes and downstream signaling components [27]. These lncRNAs are involved in metabolite biosynthesis and signal transduction by promoting the expression of target genes and suppressing specific miRNAs [26].

Human selection has played a pivotal role in driving significant phenotypic changes in crops, setting them apart from their wild ancestors. The genetic mechanisms underlying these changes have offered valuable insights for crop breeding and improvement [28, 29]. To date, extensive research based on resequencing and transcriptome data has been conducted in various crops, resulting in the identification of numerous genes that have been selected for their association with important domestication traits [30, 31, 32, 33, 34, 35]. For example, by analyzing resequencing data from 113 pear accessions, our research group has identified regions indicative of selective sweeps during the domestication processes of Asian and European pears. We have also identified selected genes linked to fruit characteristics, such as stone cell content, flesh texture, sugar content, acidity, and aroma [25]. Meanwhile, we investigated the expression variation of PCGs during pear domestication and improvement, uncovering differentially expressed genes (DEGs) associated with traits like stone cell content, fruit size, and sugar content [35]. Although lncRNAs are widely implicated in plant growth and development [36, 37], their roles in fruit crop domestication have received limited attention.

Pear (*Pyrus* spp.) exhibits remarkable phenotypic diversity, particularly in traits such as fruit size, texture, and flavor, making it an excellent model for studying fruit domestication and improvement. Among at least 22 recognized pear species worldwide, only five have been widely cultivated, while others remain as wild germplasm [34]. Notably, only the wild ancestors of *P. pyrifolia* (sand pear) and *P. ussuriensis* have been clearly identified [35]. *P. pyrifolia* is mainly distributed in southern China, where it originated and diversified, and where a broad range of germplasm—including wild progenitors, landraces, and improved cultivars—is still preserved [38]. Thus, *P. pyrifolia* serves as an ideal system for investigating pear domestication and genetic improvement.

In this study, we collected 41 *P. pyrifolia* pear accessions comprising 14 wild progenitors, 12 landraces, and 15 improved pears. We conducted genomic, transcriptome, and metabolome sequencing to investigate the expression variation of lncRNAs. Through a comparative transcriptome analysis, we observed substantial expression variation among lncRNAs, with a total of 857 and 519 lncRNAs under selection during pear domestication and improvement, respectively. These selected lncRNAs were linked to fruit size, sugar content, and stone cell content. By integrating the transcriptomic and metabolomic data, we established a lncRNA‐mRNA co‐expression network associated with lignin biosynthesis. Our study highlights the significance of artificial selection acting on lncRNAs as a mechanism contributing to changes in domestication traits in pear.

## 2 Results

### 2.1 Genome‐Wide Discovery of lncRNAs in the Pear Genome

To explore the expression dynamics of lncRNAs throughout the process of pear domestication and improvement, we conducted ssRNA‐seq on a representative assortment of 41 sand pear accessions (*P. pyrifolia*) (Table S1). This collection comprises 14 wild pears, 12 landraces, and 15 improved pears (Figure S1). Previous studies have shown that lncRNAs exhibited high tissue‐ and stage‐specificity [39, 40]. Therefore, to capture a more comprehensive set of lncRNAs, we additionally sequenced young leaves and fruits from two key developmental stages (the young [50 days after flowering] and enlarged [90 days after flowering] fruit periods) across nine representative pear accessions. Our efforts yielded an extensive dataset of approximately 3.5 terabytes of ssRNA‐seq data (Table S2). After quality filtering, 85%–95% of the reads mapped to the ‘Cuiguan’ reference genome, indicating the overall high quality of the sequencing data [41] (Table S3 and Figure S2A).

We used a modified computational pipeline to identify lncRNAs (Figure 1A). Initially, we de novo assembled a total of 190 683 transcripts using Cufflinks [42], which overlapped with 94% of known PCGs from the reference genome (Figure 1B). Subsequently, we eliminated 227 transcripts with a length of less than 200 nucleotides. By assessing the positional relationship between transcripts and annotated protein‐coding genes (PCGs), we excluded transcripts overlapping annotated PCGs on the same strand, resulting in 37 900 retained transcripts classified as class codes “i,” “x,” and “u,” representing intronic, antisense, and intergenic transcripts, respectively (Figure 1C). We then removed lowly expressed transcripts, using FPKM thresholds of ≤ 0.5 for multi‐exon transcripts and ≤ 2 for single‐exon transcripts. This process left us with 22 982 transcripts originating from 13 278 loci. Finally, we assessed the coding potential of these transcripts using various tools, such as CNCI, CPC2, CPAT, PLEK, and Pfam (Figure 1D), which led to the identification of 12 422 candidate lncRNAs originating from 8876 loci within the pear genome. Leaf and young fruit samples exhibited more lncRNAs in landrace and improved groups, and leaf, young fruit, and enlarged fruit samples exhibited more lncRNAs in the wild group (Figure S3). Among 12 422 candidate lncRNAs, 7499 lncRNAs were previously known in apple, as recorded in PLncDB, and 4923 were novel lncRNAs (Figure S2B). Based on their positional relationship compared to nearby PCGs (Figure S2C), lncRNAs were divided into three classes: 10 940 lincRNAs, 918 NATs, and 564 incRNAs (Figure 1E,F and Table S4).

**FIGURE 1:**
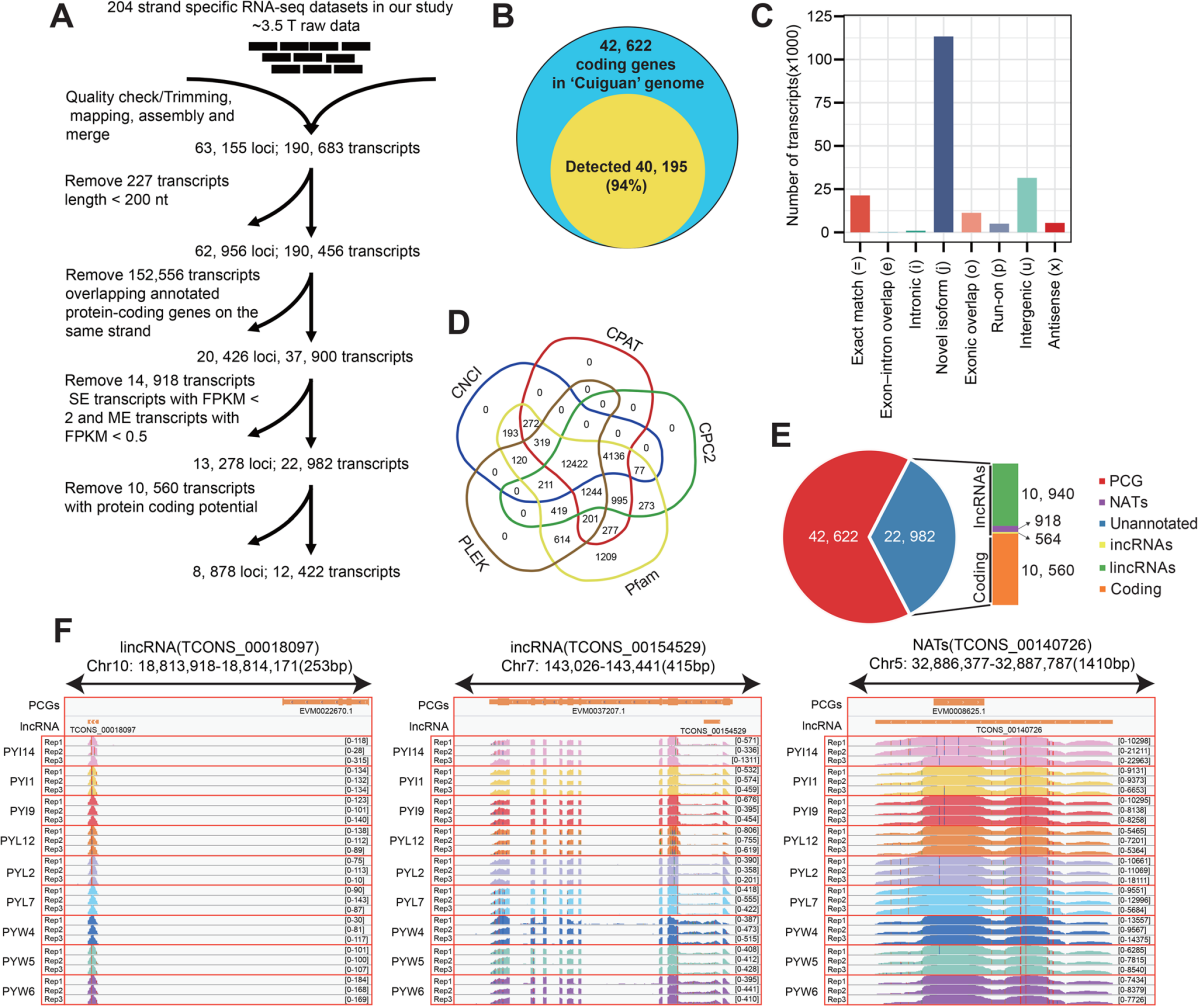
The genome‐wide identification of lncRNAs in the pear genome. (A) The pipeline of lncRNA identification, including the three main processes: transcript assembly, base filtering (length, chromosome location, and expression level), and protein coding potential prediction. (B) The count of overlapping transcripts between PCGs found in the reference genome and the 190 683 transcripts newly reconstructed using Cufflinks software is as follows: our transcript dataset successfully recaptured or overlapped with 94% of the known PCGs. (C) The classification of transcripts based on location relationship with protein‐coding genes. “=” represents complete match of intron chain; “e” represents a single exon transfrag overlapping a reference exon and at least 10 bp of a reference intron; “i” represents a transfrag falling entirely within a reference intron; “j” represents a potentially novel isoform (fragment) where at least one splice junction is shared with a reference transcript; “o” represents a generic exonic overlap with a reference transcript; “p” represents a possible polymerase run‐on fragment (within 2 kb of a reference transcript); “u” represents an unknown, intergenic transcript; “x” represents exonic overlap with a reference on the opposite strand. (D) Five methods (CPC2, CNCI, Pfam, PLEK, and CPAT) were used to predict the coding potential of candidate lncRNAs. (E) The classification of lncRNAs based on the location relationship between lncRNAs and protein‐coding genes. The pie chart represents the numbers of lincRNAs, incRNAs, and NATs. (F) Representative examples of three types of lncRNAs (lincRNA, *TCONS_00018097*; incRNA, *TCONS_00154529*; NAT, *TCONS_00140726*). Expression in nine different pear accessions (three biological replicates) is shown. Note that the *y*‐axis scales differ among samples to facilitate visualization of internal read coverage patterns and lncRNA transcript structures and are not intended for quantitative comparison of expression levels across samples.

### 2.2 TEs Embedded in lncRNAs Contribute to Sequence and Expression Variation of lncRNAs

In comparison to PCGs, lncRNAs showed a nonuniform genomic distribution across pear chromosomes (Figure 2A and Figure S4A). To further characterize this pattern, we performed a window‐based analysis across the pear genome. We found that lncRNA abundance was positively correlated with TE abundance, whereas no significant association was detected between lncRNA abundance and gene density. In addition, gene density was negatively correlated with TE abundance (Figure S4B). These results indicate that lncRNAs tend to be associated with TE‐rich regions.

**FIGURE 2:**
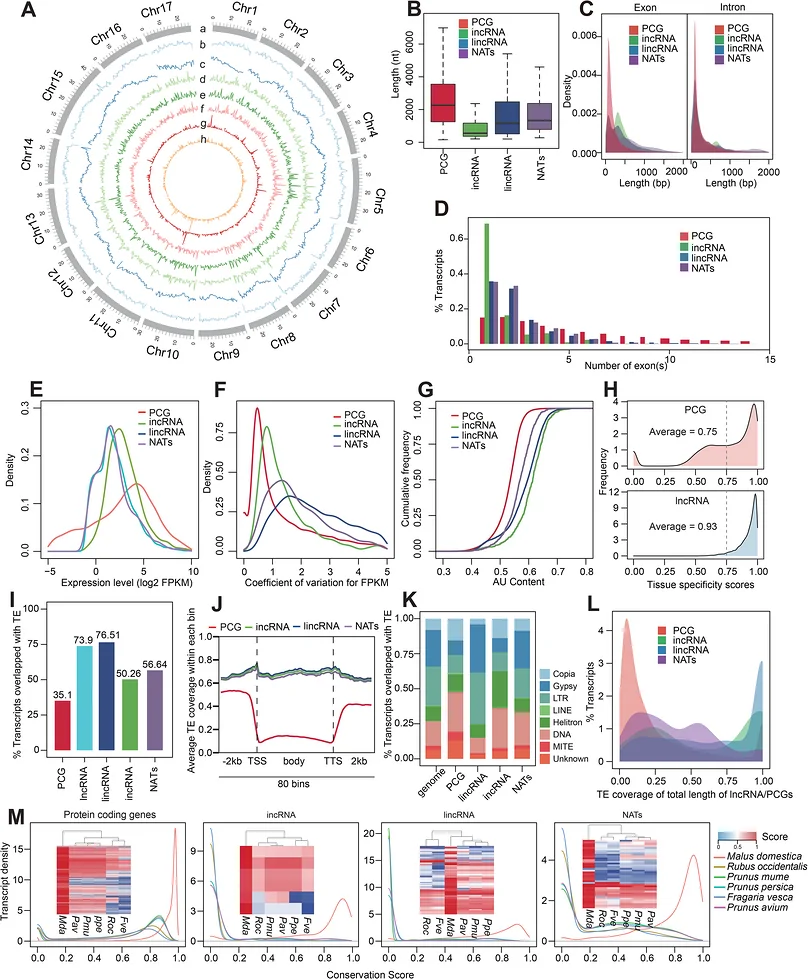
The feature analysis of lncRNAs. (A) The distribution of lncRNAs and PCGs across 17 pear chromosomes. (a) 17 pear chromosomes (b) distribution of TE density (window size, 500 kb). (c) The density of PCGs. (d) The density distribution of lncRNAs. (e) Density distribution of TE‐lncRNAs. (f) The distribution of lincRNAs. (g) The density distribution of NATs. (h) The density distribution of incRNAs. (B) The comparison of transcript length of lincRNA, incRNA, NATs, and PCG. The sample sizes were *n* = 42 622, 10 940, 564, and 918 for PCGs, lincRNAs, incRNAs, and NATs, respectively. lncRNAs generally have shorter transcript lengths than PCGs. (C) The comparison of exon and intron length among lincRNAs, incRNAs, NATs, and PCGs. The exon and intron lengths of lncRNAs are relatively longer than PCGs. (D) The comparison of exon number between lincRNAs, incRNAs, NATs, and PCGs. LncRNAs contained a lower number of exons compared to PCGs. (E) The comparison of expression levels (log_2_[FPKM]) between lincRNAs, incRNAs, NATs and PCGs. LncRNAs exhibited lower expression levels than protein‐coding genes. (F) The comparison of CV for FPKM between lincRNAs, incRNAs, NATs and PCGs. The CV values of lncRNAs were higher than PCGs. (G) The comparison of AU content between lincRNAs, incRNAs, NATs and PCGs. LncRNAs showed higher AU content than protein‐coding genes. (H) The comparison of tissue specificity scores between lncRNAs and PCGs. (I) The proportion of TE‐overlapped lncRNAs and TE‐overlapped PCGs. The proportion of TE‐overlapped lncRNAs was significantly higher than that of PCGs. (J) TEs density distribution across lncRNA/PCG body, 2 kb up‐ and downstream. The *y*‐axis represents the average TE coverage within each bin, and the *x*‐axis reflects the relative position from upstream 2k to downstream 2k of the transcript. (K) Distribution of TEs in different gene/lncRNA features (upstream, exon, intron, and downstream regions). The Gypsy and Copia families exhibited the highest proportion of TEs in lncRNAs and protein‐coding genes. (L) TE contribution proportion in lncRNAs/PCGs. (M) Sequence conservation of pear lncRNAs/PCGs across six Rosaceae species, including *Malus domestica*, *Rubus occidentalis*, *Prunus mume*, *Prunus persica*, *Fragaria vesca*, and *Prunus avium*. The distribution of sequence conservation scores is shown for PCGs, incRNAs, lincRNAs, and NATs. The heatmap represents sequence conservation scores of lncRNAs/PCGs across six Rosaceae species.

In pear, the full‐length of lncRNA transcripts tends to be on average shorter than that of PCGs (Figure 2B). However, lncRNAs displayed longer exons and introns compared to PCGs (Figure 2C). Another noteworthy difference was that lncRNAs typically contained a lower number of exons, with approximately 37% of lncRNAs being mono‐exonic, in contrast to only 15% of PCGs (Figure 2D). Furthermore, lncRNAs exhibited relatively lower expression levels than PCGs (Figure 2E) (two‐sided unpaired Student's *t*‐test, *p*‐value = 1.2 × 10^−13^). Additionally, the expression variation, as measured by the coefficient of variation (CV), among lncRNAs (median CV = 2.51) was significantly greater than that observed in PCGs (median CV = 0.98) (Figure 2F) (two‐sided unpaired Student's *t*‐test, *p*‐value < 2.2 × 10^−16^). LincRNAs (median CV = 2.67) exhibited the highest expression variation, followed by NATs (median CV = 1.78) and incRNAs (median CV = 1.15). These results are consistent with the results in rice [21] and *Arabidopsis* [43] that the expression variation of lncRNAs is substantially greater than that of mRNAs. Notably, pear lncRNAs displayed higher AU content, with an average of 58.40%, in contrast to PCGs (average: 52.85%). This trend in AU content is consistent with findings in *Arabidopsis*, rice and animal lncRNAs [44, 45, 46] (Figure 2G).

It has been postulated that lncRNAs exhibit greater tissue specificity compared to PCGs [47, 48]. To assess this hypothesis in the context of pear, we calculated the tissue specificity scores for lncRNAs and PCGs across various tissues and fruit development stages. We found that a significant majority of lncRNAs (approximately 96.03%) displayed tissue specificity scores exceeding 0.75, whereas only 59.49% of PCGs exhibited such high scores (Figure 2H). These findings strongly support that lncRNAs in pear indeed exhibit higher tissue‐specificity than PCGs.

Our analysis revealed that a substantial proportion of lncRNAs, approximately 74%, contained transposable elements (TEs), which was significantly higher than the proportion found in PCGs, standing at 35.1% (Figure 2I). TE lncRNA set contained 8415 lincRNAs, 291 incRNAs, and 521 NATs, such as the NAT *TCONS_00004201* (Figure S5). Notably, among lncRNAs, lincRNAs had the highest proportion of TEs (76.51%). This observation aligns with the distribution patterns of TEs, which tend to preferentially reside away from genes but show a high enrichment within lncRNAs (Figure 2J). Furthermore, we examined the distribution of different types of TEs within lncRNAs and PCGs. Among the lncRNAs containing TEs, retrotransposons were more prevalent than DNA transposons. Specifically, 26.8% and 19.9% of lncRNAs were found to harbor LTR and Gypsy type TEs, respectively (Figure 2K). Additionally, transcripts that contained TEs contributed a larger portion of their sequences to lncRNAs, with an average lncRNA coverage by TEs of 64.31%, compared to an average of 25.59% for PCGs (Figure 2L). This pattern aligns with previous observations in the human, mouse, and zebrafish genomes [49]. Only a small subset of lncRNAs showed complete coverage by TEs, with a coverage ratio approaching 1.0, whereas the majority showed either partial or no overlap with TE. Notably, when compared to lncRNAs lacking TEs, lncRNAs overlapping with TEs exhibited lower expression levels, higher tissue specificity scores, longer transcript lengths, and increased expression variation (two‐sided unpaired Student's *t*‐test, *p*‐value < 2.2 × 10^−16^) (Figure S6A–D). Further subclass‐specific analysis showed that LTR‐, Gypsy‐, and other TE‐associated lncRNAs followed the same overall trend as TE‐associated lncRNAs as a whole, exhibiting higher CV, longer transcript length, higher tissue specificity, and lower expression levels than non‐TE‐associated lncRNAs (Figure S6E–H). These findings suggest that TEs embedded within lncRNAs may have a significant impact on their sequence composition, expression variation, and tissue‐specific expression.

The conservation of lncRNAs can provide valuable insights into predicting their potential functions. In our analysis, we aligned lncRNAs from pear to six representative Rosaceae genomes using GMAP [38]. We used the sequence coverage and identity of the aligned results to calculate a conservation score. We found a prominent peak in the conservation score distribution of lncRNAs clustered near zero. This suggests that the sequence conservation of lncRNAs in Rosaceae is relatively poor (Figure 2M). However, when we examined PCGs, we noticed peaks in their conservation score distribution falling within the range of 0.8–1.0. This indicates that the sequence conservation of PCGs in Rosaceae is notably higher compared to lncRNAs. Among three types of lncRNAs, NATs exhibited a higher degree of sequence conservation compared to lincRNAs and incRNAs (Figure S7).

To further characterize the potential regulatory relationships between lncRNAs and protein‐coding genes, we identified both cis‐ and trans‐regulated lncRNA–mRNA pairs. Protein‐coding genes located within 10 kb upstream or downstream of each lncRNA were defined as potential cis target genes, resulting in 20 858 putative cis lncRNA–mRNA pairs. In parallel, putative trans‐regulated pairs were identified based on expression correlation analysis across samples using the criteria of |PCC| > 0.6 and *p* < 0.001, which yielded 315 639 putative trans lncRNA–mRNA pairs. Among the trans pairs, 195 035 (61.79%) showed positive correlations, whereas 120 604 (38.21%) showed negative correlations.

We further investigated the potential role of lncRNAs as endogenous target mimics (eTMs) in pear. A total of 405 putative lncRNA–miRNA eTM pairs, involving 395 lncRNAs and 66 miRNAs, were identified. By integrating predicted miRNA targets, we constructed a putative lncRNA–miRNA–mRNA regulatory network containing 18 424 tripartite relationship pairs (Figure S8), suggesting that certain lncRNAs may participate in post‐transcriptional regulation through interactions with miRNAs and their target genes.

### 2.3 DNA Methylation is Associated with Expression Variation in Approximately 32% of lncRNAs

DNA methylation is an important epigenetic modification. To investigate the relationship between DNA methylation and lncRNA expression, we compared the DNA methylation profiles of lncRNAs and mRNAs. LncRNAs exhibited relatively low DNA methylation levels around the transcription start site (TSS), a pattern similar to that observed for PCGs (Figure 3A). However, lncRNAs exhibited relatively high DNA methylation levels around the transcription termination site (TTS), resembling the pattern observed for TEs. When comparing the overall patterns of DNA methylation levels, we found that lincRNAs exhibited significantly higher DNA methylation levels than that of PCGs in any region, including upstream 2k, body, and downstream 2k (Figure 3B and Figure S9). To test the effects of DNA methylation on lncRNA expression, we divided all lncRNAs into four groups (low, mid‐low, mid‐high, and high) based on their expression levels. The DNA methylation levels of the four groups in the upstream 2‐kb, gene body, and downstream 2‐kb sequences of the lncRNAs were compared. The high‐expression group exhibited the lowest methylation levels in the 2 kb upstream region, body, and 2 kb downstream region among the four groups. The CG and CHG methylation at the lncRNA body was negatively correlated with lncRNA expression levels. Previous studies had reported that gene expression level was negatively correlated with DNA methylation level of upstream2k regions of genes [50, 51]. However, there are no obvious correlations between lncRNA expression level and methylation located on upstream2k and downstream2k region of lncRNA (Figure 3C).

**FIGURE 3:**
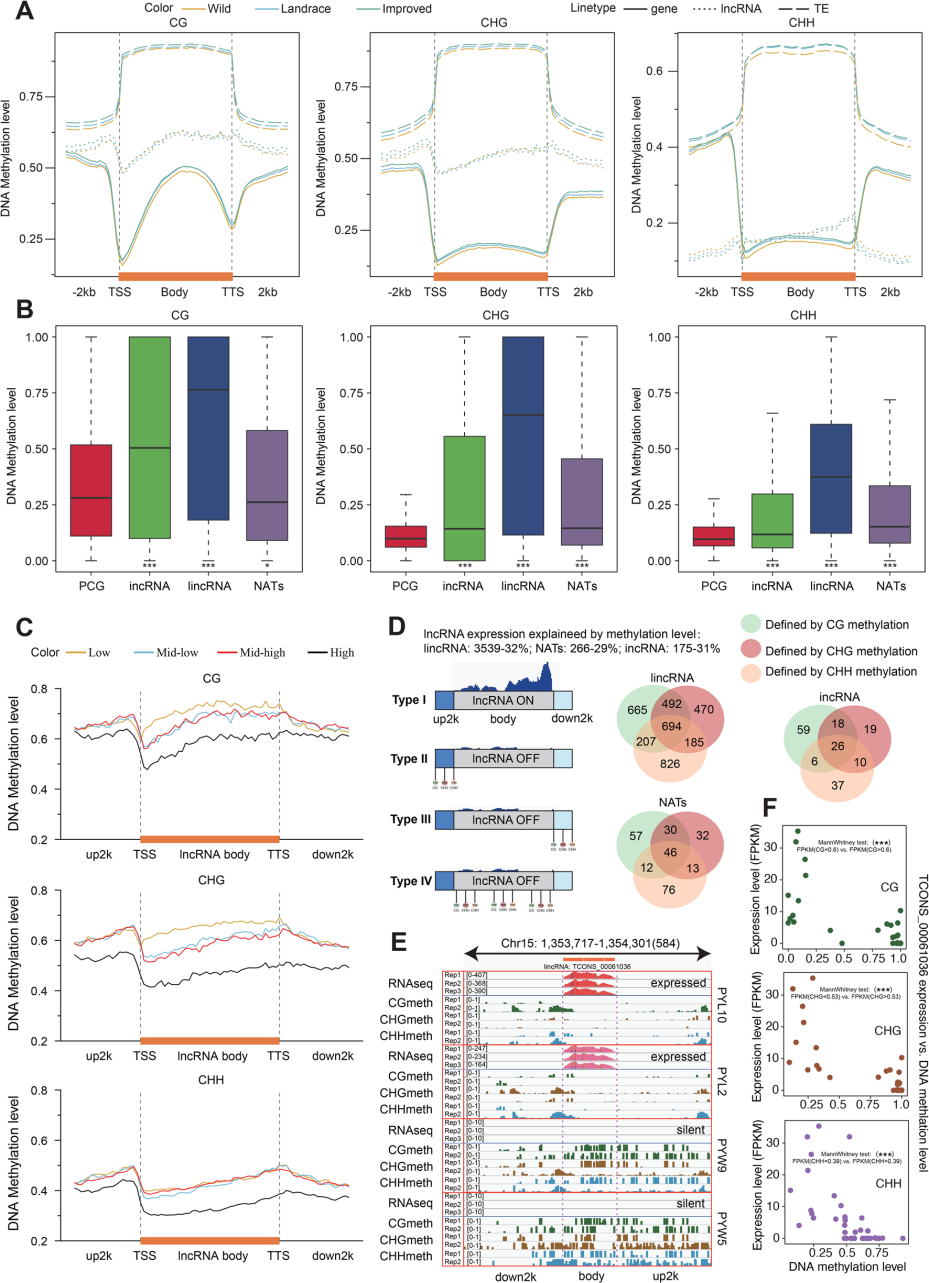
The epigenetic regulation of lncRNAs. (A) Distribution of DNA methylation levels across the upstream 2 kb, gene body, and downstream 2 kb regions of lncRNAs, PCGs, and TEs. (B) DNA methylation level at gene body regions of PCGs, incRNAs, lincRNAs, and NATs across 41 pear accessions. The sample sizes were *n* = 42 622, 10 940, 564, and 918 for PCGs, lincRNAs, incRNAs, and NATs, respectively. *p* values were calculated using the Mann–Whitney U test: *p < 0.05, ***p < 0.001. (C) Relationship between CG, CHG and CHH methylation levels and expression levels for all lncRNAs in the 2 kb upstream, lncRNA body, and 2 kb downstream regions. (D) Summary of lncRNAs for which expression can be explained by methylation. Type I–IV indicate different methylation configurations across the upstream 2 kb, gene body, and downstream 2 kb regions that are associated with lncRNA ON or OFF states. The Venn diagrams show the overlap between loci for lincRNAs, incRNAs, and NATs that were found to be defined by CG, CHG, or CHH methylation levels. (E) An example of lincRNA (*TCONS_00061036*) defined by CG, CHG, or CHH methylation, showing RNA‐seq signal, CG, CHG, and CHH methylation levels in four accessions. (F) Expression level as a function of CG, CHG, and CHH methylation for the example lincRNA (*TCONS_00061036*) across 41 pear accessions. The results of the Mann–Whitney U tests used for defining the explanatory power of CG, CHG and CHH methylation are shown (***p < 0.001).

To test whether DNA methylation variation can explain expression variation of lncRNAs, we utilized expression data and methylation data from 41 pear accessions. We found that the expression of 3539 lincRNAs, 266 NATs, and 175 incRNAs could be explained by the level of CG or CHG or CHH methylation at their upstream2k, body, downstream2k (Figure 3D, Methods). These numbers correspond to 32%, 29%, and 31% of all lincRNAs, NATs, and incRNAs, respectively. For genes, we could explain expression variation by variation in DNA methylation for 28% of all genes (Figure S10A). Two examples of such a lncRNA (TCONS_00061036) and a gene (*EVM0022826.1*) with high variation between accessions are displayed (Figure 3E and Figure S10B–D). The lncRNA TCONS_00061036 was highly expressed in accessions (PYL2 and PYL10) that lacked CG, CHG, and CHH methylation in the locus, but was silent in accessions (PYW5 and PYW9) which enrich CG, CHG, and CHH methylation in the locus. A very strong association was found at the locus between high methylation and low expression, or between low methylation and high expression (Figure 3F).

In summary, we determined that lncRNAs exhibited distinct DNA methylation patterns compared to protein‐coding genes, and that higher methylation levels tend to repress lncRNA expression. DNA methylation plays an important role in regulating lncRNA expression, particularly within the lncRNA body regions, and explains the variation in expression of ∼32% of all lncRNAs. Genome‐wide distribution analysis further showed that DNA methylation‐associated lncRNAs were distributed across all 17 chromosomes without obvious clustering and were highly associated with TE sequences (Figure S11).

To further explore the relationship between DNA methylation and tissue specificity of lncRNAs, we compared tissue‐specificity scores between methylation‐associated lncRNAs and non‐methylation‐associated lncRNAs. Methylation‐associated lncRNAs showed significantly higher tissue‐specificity scores than non‐methylation‐associated lncRNAs (Figure S12), suggesting that DNA methylation may contribute to the establishment or maintenance of tissue‐specific expression patterns of lncRNAs.

### 2.4 Divergence of lncRNA Expression During Pear Domestication and Improvement

To investigate the expression divergence of lncRNAs among wild, landrace, and improved pears, we conducted a principal component analysis (PCA) using the expression matrix of lncRNAs. The result of the analysis revealed that the improved pear population was distinct, while wild and landrace pears were intermingled (Figure 4A and Figure S13). This pattern suggests that lncRNA expression divergence was more pronounced during pear improvement than during domestication. We have identified differentially expressed lncRNAs (DELs) and genes (DEGs) in three comparison groups: wild‐vs.‐landrace, wild‐vs‐improved, and landrace‐vs.‐improved (Figure S14, Tables S5 and S6). Among the findings, a total of 1807 lncRNAs showed significant differential expression between wild and landrace pears, constituting 14.55% of all lncRNAs (Figure 4B). Gene Ontology (GO) enrichment analysis of the target genes (4912) of these DELs revealed that the DELs identified in the wild‐vs.‐landrace comparison were associated with the “response to abiotic stimulus” (Figure S15). Similarly, we identified 1828 DELs between landrace and improved pear varieties. Interestingly, a greater number of DELs were downregulated during both domestication and improvement. Additionally, we found that around 10% (450) and 20% (1078) of target genes of DELs displayed differential expression during pear domestication and improvement processes, respectively (Figure S15E). These findings suggest that the processes of pear domestication and improvement have dramatically reshaped the expression patterns of both lncRNA and PCGs. PCA based on these differentially expressed predicted target genes of DELs also showed clear separation between wild and landrace accessions in the domestication comparison, and between landrace and improved accessions in the improvement comparison (Figure S16). These results suggest that the predicted target genes of DELs may contribute to the transcriptional divergence during pear domestication and improvement.

**FIGURE 4:**
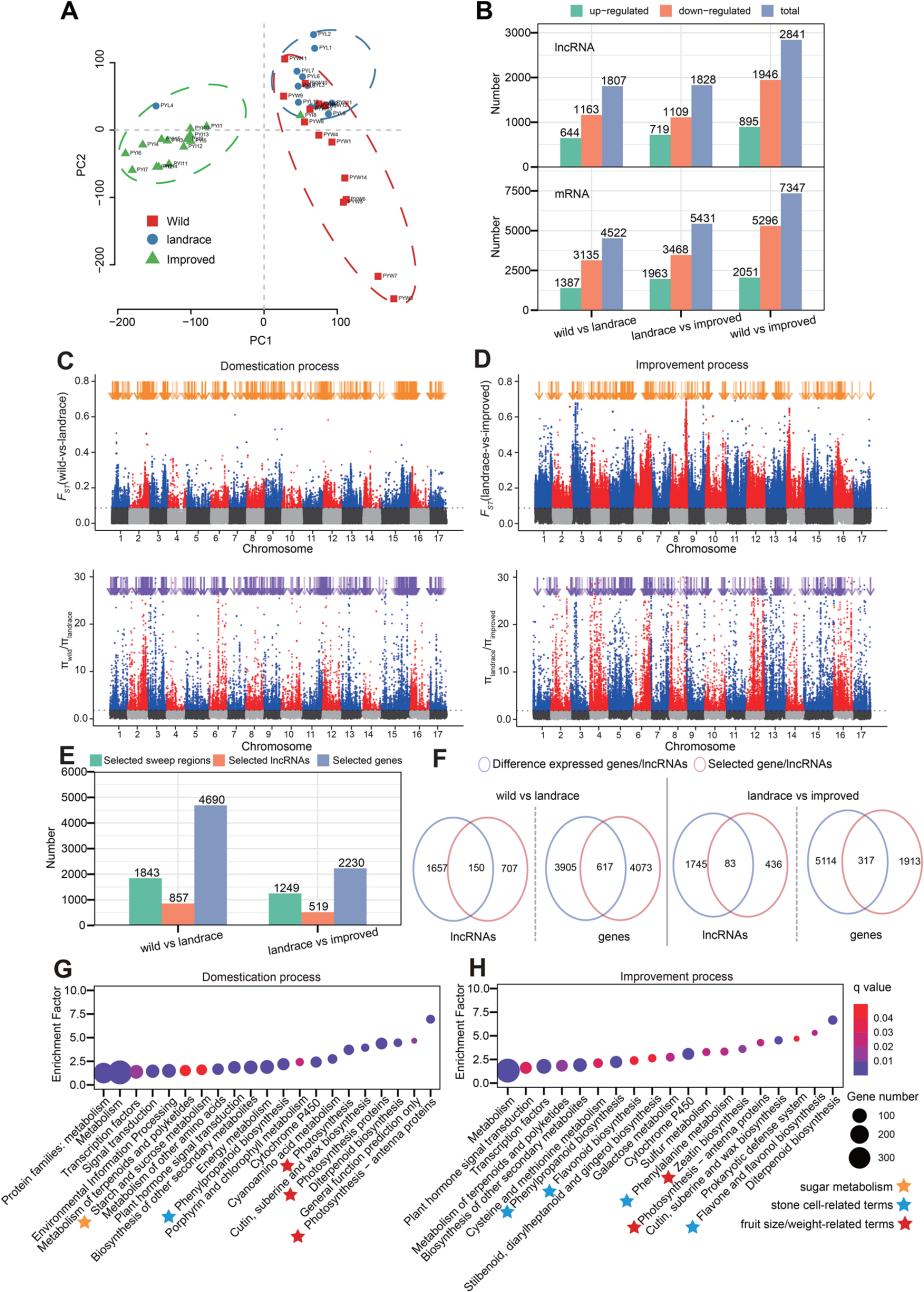
Differentially expressed lncRNAs and selection signals during pear domestication and improvement. (A) Principal variance component plots of expression levels of lncRNAs in wild, landrace, and improved populations. (B) The total number of differentially expressed lncRNAs, up‐regulated and down‐regulated lncRNAs from three comparisons: wild‐vs.‐landrace, landrace‐vs.‐improved, and wild‐vs.‐improved. The identification of selective sweep regions during pear domestication (C) and improvement process (D) based on π ratio and *F_ST_*. The regions with the top 10% *F_ST_* value and top 10% π ratio (π_wild_/π_landrace_; π_landrace_/π_improved_) were considered selective sweep regions. (E) The number of selected lncRNAs and genes during pear domestication and improvement. (F) Venn diagrams of differentially expressed lncRNAs and selected lncRNAs, and differentially expressed mRNAs and selected mRNAs. KEGG enrichment analysis of target genes of selected lncRNAs with differential expression during pear domestication (G) and improvement (H).

### 2.5 The Selection Signal on lncRNAs During Pear Domestication and Improvement

To identify lncRNAs that underwent selection during pear domestication and improvement, we analyzed the resequencing data from these 41 pear accessions and identified selective sweep regions. A total of 5 953 806 high‐quality single nucleotide polymorphisms (SNPs) were identified across 41 accessions. Based on these SNPs, we performed PCA, which showed a clear separation between wild and improved groups, whereas the landrace accessions clustered closer to the wild group. The PCA results suggest that the wild and landrace accessions share more similar genetic backgrounds compared to the improved accessions. In addition, a wider distribution of the wild group was observed in the PCA plot, suggesting that the wild and landrace groups exhibited higher diversity compared with the improved group (Figure S17A). Moreover, a maximum‐likelihood phylogenetic tree showed that the accessions of the improved group clustered together as a branch, while the wild and landrace groups formed three separate branches (Figure S17B). Population structure analysis (K = 2‐4) further indicated that the improved pear accessions exhibited a higher level of admixture than the wild and landrace groups, likely due to recent hybridizations during pear improvement (Figure S17C). These results align with transcriptome data findings (Figure 4A).

We further identified selective sweeps using the SNP data. In the domestication process, we identified a total of 1843 selective sweep regions (π_wild_/π_landrace_ ≥ 1.81; *F_ST_* [wild‐vs.‐landrace] ≥ 0.09), which collectively accounted for 41 Mb of the genome (Figure 4C), encompassing 857 lncRNAs and 4690 mRNAs (Table S7). In the improvement process, we identified 1249 selective sweep regions (π_landrace_/π_improved_ ≥ 2.38; *F_ST_* [landrace‐vs.‐improvement] ≥ 0.18), spanning 22 Mb of the genome (Figure 4D). In comparison to the domestication event, fewer lncRNAs (519) and mRNAs (2230) underwent selection during pear improvement (Figure 4E and Table S8). To examine whether the selective signals detected for lncRNAs were mainly driven by linkage to nearby protein‐coding genes, we performed a distance‐controlled analysis by stratifying lncRNAs according to their genomic distance to the nearest PCG (<5 kb, 5–20 kb, and >20 kb). Within each distance bin, selected lncRNAs consistently exhibited significantly higher π ratio values than randomly sampled non‐selected lncRNAs (Figure S18). These results suggest that the selective signals associated with selected lncRNAs cannot be fully explained by physical linkage to nearby PCGs. We note that only 150 lncRNAs and 617 mRNAs that underwent selection during domestication displayed differential expression. Likewise, only 83 lncRNAs and 317 mRNAs under selection during improvement exhibited differential expression (Figure 4F).

To further investigate the function of selected lncRNAs with differential expression, we identified the target genes of these lncRNAs and conducted Kyoto Encyclopedia of Genes and Genomes (KEGG) enrichment analysis based on these targets. The selected lncRNAs, which exhibited differential expression in both domestication and improvement processes, were notably associated with photosynthesis (Figure 4G,H). These findings suggest that selection on lncRNAs may be associated with increased fruit size and weight during pear domestication and improvement [52, 53, 54]. Another key term shared in both domestication and improvement was “phenylalanine metabolism.” Phenylalanine serves as the synthetic precursor for lignin and stone cell formation. These results imply that lncRNAs might play a role in the domestication and improvement of stone cell content in pear fruit. Additionally, “Starch and sucrose metabolism” was observed during the pear domestication process, indicating that the selection on lncRNAs is associated with increasing sugar content during pear domestication (Figure 4G,H).

### 2.6 Reduced Accumulation of Lignin‐Related Metabolites During Pear Domestication and Improvement

To investigate how expression changes of the transcriptome contribute to metabolomic variation during domestication and improvement, we carried out metabolic profiling of 41 pear fruit samples. We detected a total of 2576 metabolites by constructing a pear fruit MS2 spectral tag (MS2T) library, with 517 of these metabolites being annotated in a database and categorized into 14 different classes (Figure 5A).

**FIGURE 5:**
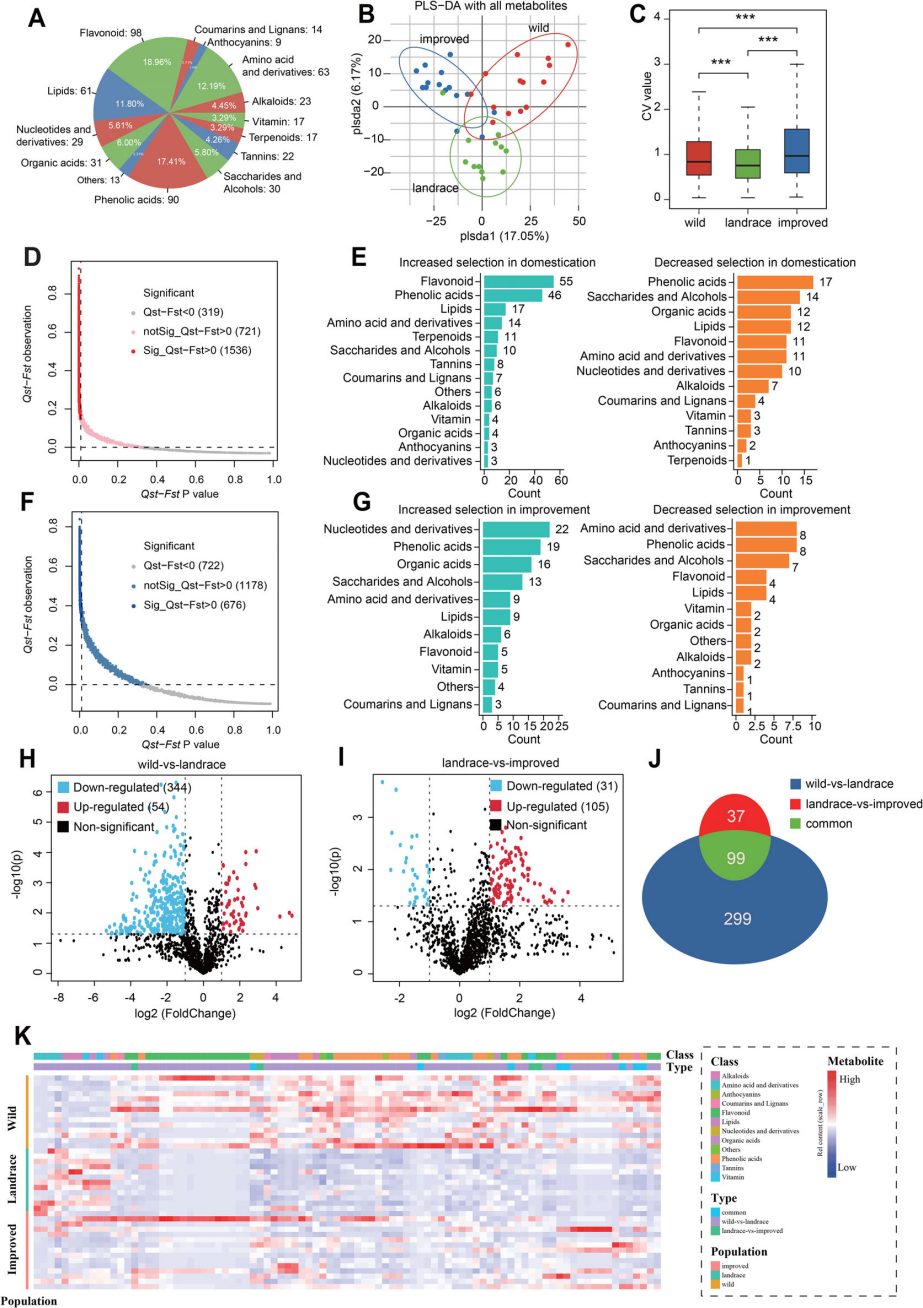
Metabolome divergence between wild, landrace and improved pears. (A) The classification of 517 annotated metabolites divided across 14 classes. (B) PLS‐DA of wild, landrace and improved pears with all 2576 metabolites. (C) Comparison of coefficient of variation (CV) values across all metabolites among wild, landrace, and improved pear groups. Statistical significance was assessed using two‐sided unpaired Student's *t*‐tests. The sample sizes were *n* = 14, 12, and 15 for wild, landrace, and improved pears, respectively. Asterisks indicate statistical significance (***p < 0.001). (D) The identification of selected metabolites during pear domestication by *Qst*‐*Fst*. (E) In the domestication process, the classification of selected metabolites shows high accumulation in wild pears (left) and landraces (right). (F) The identification of selected metabolites during the pear improvement process by *Qst*‐*Fst*. (G) In the improvement process, the classification of selected metabolites shows high accumulation in landraces (left) and improved pears (right). Volcano plot of fold change in metabolites between wild‐vs.‐landrace pears (H) and landrace‐vs.‐improved pears (I). (J) Venn diagram showing the number of differentially accumulated metabolites in two comparisons: wild‐vs.‐landrace, landrace‐vs.‐improved. (K) The heatmap represents differentially accumulated metabolites in 41 pears.

We used Partial Least Squares Discriminant Analysis (PLS‐DA) to cluster the 41 accessions based on the 2576 metabolites. Notably, the 41 pear accessions were divided into three distinct groups: wild, landraces, and improved (Figure 5B). These findings suggest that wild, landrace, and improved pears exhibit clear metabolome differences. Landraces appear to be situated between wild and improved pears, indicating a gradual evolutionary process from wild to landrace and from landrace to the improved population. Similar results were observed when using 517 annotated metabolites in the PLS‐DA analysis (Figure S19A).

We computed the coefficient of variation (CV) for metabolites in three distinct groups. Our findings revealed a 12.24% reduction in the diversity of metabolite accumulation in landrace pears compared to the wild population (two‐sided unpaired Student's *t*‐test, *p*‐value = 2.22e‐16). In contrast, there was a substantial 36.05% increase in accumulation diversity in improved pears compared to landrace pears (two‐sided unpaired Student's *t*‐test, *p*‐value = 2.22e‐16) (Figure 5C and Figure S19B). These trends were consistent across 11 out of 13 metabolite categories, including Organic acids, Saccharides, Alcohols, and Tannins. However, Anthocyanins and Terpenoids showed a consistent increase in accumulation diversity during pear domestication and improvement (Figure S19C).

We employed the quantitative trait differentiation (*Q* *st*) and fixation index (*Fst*) strategy to identify metabolites that underwent direct selection throughout the course of pear domestication and improvement. A total of 1536 metabolites exhibited potential directional selection during pear domestication (*p* < 0.01) (Figure 5D). This set comprised 937 metabolites that showed higher accumulation in landrace pears and 599 metabolites that displayed a decreasing pattern from wild to landrace pear. Notably, five metabolites related to lignin demonstrated reduced levels in landrace pears, specifically p‐Coumaric acid, p‐Coumaryl alcohol, Sinapin aldehyde, Coniferyl alcohol, and Coniferyl aldehyde. Additionally, seven Coumarin and lignan metabolites exhibited a decrease in accumulation in landrace pears (Figure 5E). In comparison to the domestication process, fewer metabolites (676, 26.24%) were selected during the improvement process (Figure 5F). We also observed that five lignin‐related metabolites showed reduced accumulation during the improvement process, including p‐Coumaraldehyde, Isochlorogenic acid A, Coumaric acid‐glucoside, Caffeic acid, and Chlorogenic Acid (Figure 5G).

MetaboAnalyst 5.0 tools were employed to detect differentially expressed metabolites (DEMs). We identified 398 DEMs during pear domestication and 136 DEMs during pear improvement (Figure 5H,I, Figure S19D, and Table S9). These findings highlight that the process of domestication induced significant alterations in pear fruit metabolites compared to recent improvement efforts. Additionally, we found 99 (22.76%) DEMs that underwent selection during both domestication and improvement processes, indicating their importance as targets for pear breeding (Figure 5J). Notably, eight metabolites associated with lignin biosynthesis exhibited a decrease during both pear domestication and improvement (Figure 5K).

### 2.7 Construction of lncRNA‐mRNA Co‐Expression Modules for Functional Prediction of lncRNAs

To uncover the genetic mechanisms responsible for changes in metabolites during pear domestication and improvement, we employed weighted gene co‐expression network analysis (WGCNA) to construct lncRNA‐mRNA co‐expression modules. Ultimately, we generated 52 co‐expression modules using a soft‐thresholding power (β = 5) (Figure 6A and Figure S20).

**FIGURE 6:**
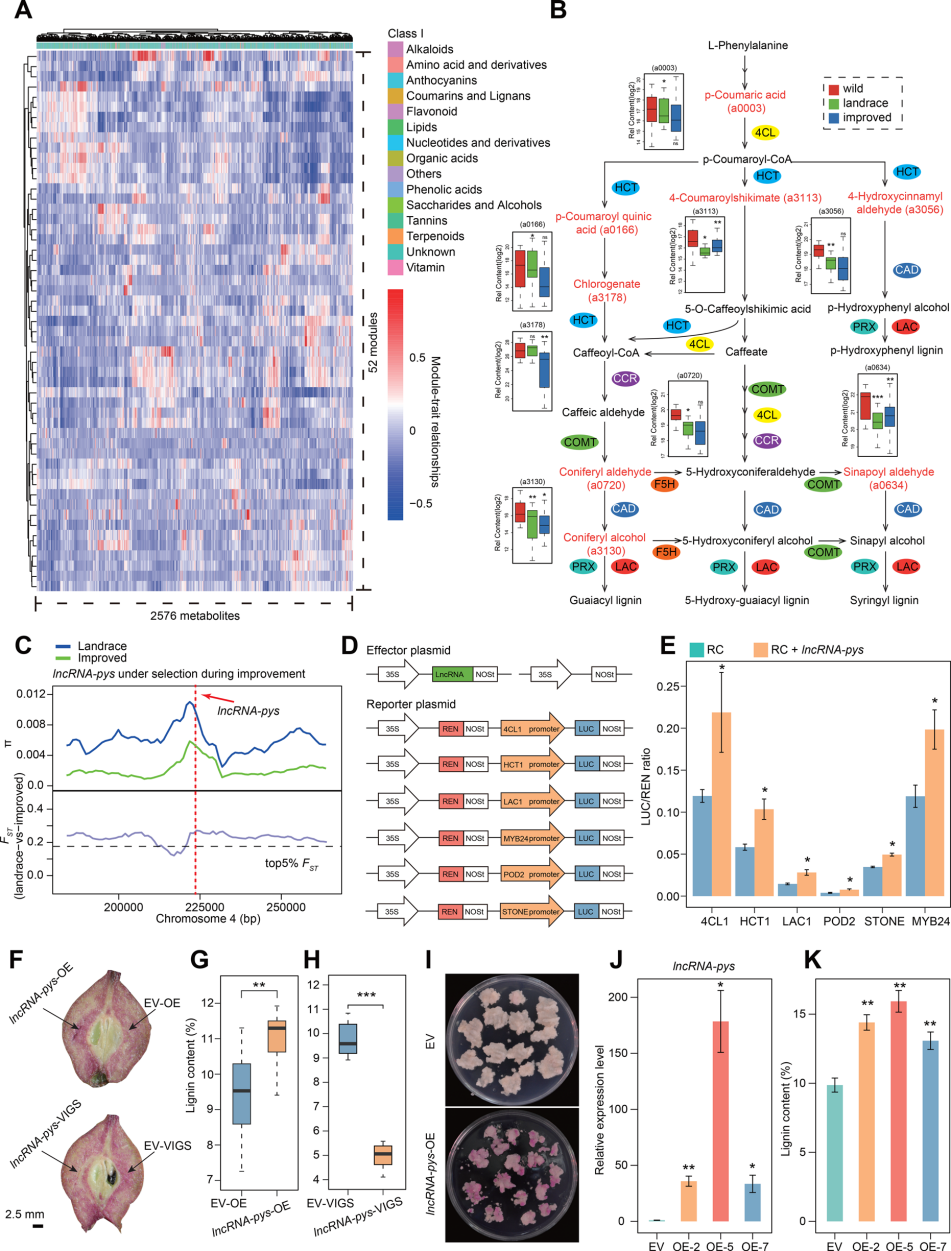
The construction of lncRNAs‐mRNAs‐metabolites co‐expression module. (A) Transcriptomic and metabolic correlation analysis during the pear domestication and improvement process using WGCNA. The heatmap represents the correlation between 52 co‐expression modules and 2576 metabolites. (B) Lignin biosynthesis pathway and evidence of metabolite divergence between wild, landrace and improved pear populations. The boxplots represent the relative metabolite content in wild (*n* = 14), landrace (*n* = 12) and improved (*n* = 15) pears. *p*‐values were tested using two‐sided unpaired Student's *t*‐tests (*p < 0.05, **p < 0.01, ***p < 0.001, ns indicates no significant difference). (C) Distribution of *F_ST_* and π on *lncRNA‐pys* during the pear improvement process. (D) Schematic representation of the LUC reporter vector containing lignin‐related gene promoters (*pro*::*LUC*), empty vector (35S), and effector vector lncRNA (*35S*::*lncRNA*). (E) Transient LUC imaging assays showing *lncRNA‐pys* activates the expression of lignin‐related genes (*pro*::*LUC*). Representative images of LUC activity in *N. benthamiana* leaves 40 h after infiltration are illustrated. The dual LUC assay shows promoter activity expressed as a ratio of promoter LUC to 35S::Renilla. Error bars represent the SD for three replicates (*n* = 3 independent biological replicates). *p*‐values were determined by computing two‐sided unpaired Student's *t*‐tests in the R software (*p < 0.05). (F) Cross‐sections of pear fruits transiently transformed with the overexpression construct (*lncRNA‐pys*‐OE) or the silencing construct (*lncRNA‐pys*‐VIGS), together with their corresponding empty‐vector controls (EV‐OE and EV‐VIGS). The infiltrated regions are indicated by arrows. Scale bar = 2.5 mm. (G) Lignin content in pear fruit tissues surrounding the infiltration sites after transient overexpression of *lncRNA‐pys* compared with the corresponding empty‐vector control (EV‐OE). (H) Lignin content in pear fruit tissues surrounding the infiltration sites after transient silencing of *lncRNA‐pys* compared with the corresponding empty‐vector control (EV‐VIGS). Data in (G) and (H) are presented as mean ± SD (*n* = 9 independent biological replicates). Statistical significance was determined using two‐sided unpaired Student's *t*‐tests (**p < 0.01, ***p < 0.001). (I) Phenotypic comparison of pear callus transformed with empty vector (EV) and overexpression construct of *lncRNA‐pys* (OE), stained with Wiesner's reagent to visualize lignin accumulation. (J) Relative expression levels of *lncRNA‐pys* in transgenic pear callus (OE‐2, OE‐5, OE‐7) measured by qRT‐PCR. (K) Lignin content in transgenic callus lines was determined using the acetyl bromide method. Data in (J) and (K) are presented as mean ± SD (*n* = 3 independent biological replicates). *p*‐values were tested using two‐sided unpaired Student's *t*‐tests (*p < 0.05; **p < 0.01).

Correlation analysis between the modules and metabolites revealed several significant associations. Specifically, the MEpink module exhibited a strong positive correlation with metabolites such as D‐(+)‐Phenylalanine (*p*‐value = 6 × 10^−4^), L‐Phenylalanine (*p*‐value = 1 × 10^−4^), Ferulic acid (*p* value = 0.03), 1‐O‐Feruloyl quinic acid (*p* value = 8 × 10^−4^), which are related to stone cell content in pear fruit (Figure 6A). Additionally, MEblue, MEsalmon4, MEnavajowhite2 and MEgrey60 modules were positively correlated with Coniferyl alcohol, while MEdarkolivegreen4 and MEdarkslateblue were positively correlated with Coniferyl aldehyde. Coniferyl aldehyde and Coniferyl alcohol are vital synthetic precursors of G‐lignin in pear. The MEdarkgrey module exhibited a positive correlation with Sinapoyl aldehyde, another crucial synthetic precursor for S‐lignin in pear. These findings suggested that lncRNAs and genes within these modules may play essential roles in stone cell synthesis. Furthermore, four modules (MEorangered3, MEblue, MElightgreen, MEskyblue1) were found to correlate with malic acid metabolism, while MEsaddlebrown and MEmaroon might be linked to sorbitol and sucrose, respectively. The lists of lncRNAs and genes in these candidate modules are provided in Table S10, offering a valuable dataset for enhancing the flavor of pear fruit in future efforts.

### 2.8 *lncRNA‐pys* Affects *STONE* Expression to Regulate Stone Cell Formation

In the process of pear domestication and improvement, there has been a significant decrease in the stone cell content of improved and landrace pears when compared to wild pears (Figure S1). Lignin constitutes the primary component of pear stone cells. Based on our metabolomic data, we observed down‐regulated patterns in many intermediate products within the lignin biosynthetic pathway during pear domestication and improvement (Figure 6B). These down‐regulated metabolites included Caffeoyl‐p‐coumaroylquinic acid, Chlorogenate, Coniferyl aldehyde, Coniferyl alcohol, p‐Coumaric acid, 4‐Hydroxycinnamyl aldehyde, 4‐Coumaroylshikimate and Sinapoyl aldehyde. In addition, a significant reduction in cellulose content was observed from wild to landrace and further from landrace to improved pears. Cellulose is one of the major components of the secondary cell wall of stone cells (Figure S21) [55].

Our WGCNA analysis revealed a positive correlation between the MEpink module and the lignin biosynthetic pathway (Figure S22). Furthermore, genes within the MEpink module were significantly enriched in terms related to the “Casparian strip” and the “secondary cell wall” (Figure S22A). Therefore, we consider the MEpink module as associated with stone cell formation. We also identified a substantial number of transcription factors involved in lignin or stone cell formation within this module, including *MYB15*, which previous studies reported contributes to lignin formation by activating lignin structural genes such as *PAL*, *C4H*, *4CL*, *HCT*, *C3’H*, *COMT* and *CAD* in *Arabidopsis* [56]. We also identified five lignin‐related structural genes within the co‐expression network, including *STONE*, *CAD4*, *CAD7*, *CCR6* and *EIF‐5A*. Among them, *STONE* had been functionally verified as playing a role in the regulation of stone cell formation, as described in our previous study [57].

Further, we established a co‐expression network that included lncRNAs, transcription factors, and structural genes associated with lignin metabolism (Figure S22B). This analysis revealed that lncRNAs and genes shared similar expression patterns, suggesting a potential regulatory relationship between them. The lncRNA *TCONS_00028382* , hereafter designated as *lncRNA‐pys*, exhibited high expression levels during the early stages of fruit development (35 DAF), followed by reduced expression in the later stages (Figure S23). This expression pattern was consistent with the substantial accumulation of lignin and stone cell content observed during early fruit development [58]. High *F_ST_* values and a reduction in π values in the genomic regions of *lncRNA‐pys* indicated that this lncRNA was under selection during improvement (Figure 6C and Table S11). We further analyzed the expression pattern of *lncRNA‐pys* across wild, landrace, and improved accessions. A significant expression decline was observed in improved accessions (Figure S24), supporting the hypothesis that this lncRNA underwent selection during pear domestication and improvement. Interestingly, we found a significantly negative correlation between the expression level of *lncRNA‐pys* and CG and CHH methylation levels at *STONE* promoter (Figure S25). These findings indicate that *lncRNA‐pys* likely reduces DNA methylation at the promoter of the *STONE* gene, thereby enhancing *STONE* gene transcription, consistent with the activation observed in dual‐luciferase reporter assays.

We performed dual‐luciferase reporter assays to assess whether *lncRNA‐pys* affects the promoter activity of lignin‐related genes. Co‐infiltration of *lncRNA‐pys* with promoter constructs of five structural genes (*STONE*, *4CL1*, *HCT1*, *LAC1* and *POD2*) and one known transcription factor (*MYB24*) in tobacco leaves revealed that *lncRNA‐pys* significantly enhanced the activity of *4CL1*, *HCT1*, *LAC1*, *STONE*, *POD2* and *MYB24* (Figure 6D,E). These findings suggest that *lncRNA‐pys* may act as a transcriptional regulator of both structural and transcription factor genes involved in lignin biosynthesis.

To verify the role of *lncRNA‐pys* in lignin accumulation in pear fruits, we generated both overexpression and silencing constructs and transiently introduced them into pear fruits by agroinfiltration. Hand‐cut sections stained with phloroglucinol‐HCl showed substantially stronger lignin staining at the sites injected with the *lncRNA‐pys* overexpression construct than at the sites injected with the corresponding empty vector control. In contrast, lignin staining was clearly weaker at the sites injected with the *lncRNA‐pys* silencing construct than at the sites injected with the corresponding VIGS empty vector control (Figure 6F). Consistently, lignin content was significantly increased by transient overexpression of *lncRNA‐pys*, whereas silencing of *lncRNA‐pys* significantly reduced lignin content (Figure 6G,H). These reciprocal results provide functional evidence that *lncRNA‐pys* positively contributes to lignin accumulation in pear fruits.

To further confirm the role of *lncRNA‐pys* in lignin accumulation in pear fruits, the empty vector (EV) and *lncRNA‐pys* overexpression constructs were stably transformed into pear callus. After phloroglucinol HCl staining, the pear fruit calli transformed with the *lncRNA‐pys* overexpression constructs exhibited a strong red color, while calli transformed with the empty vector showed very weak staining (Figure 6I). Lignin content was significantly increased in *lncRNA‐pys*‐OE callus compared to calli transformed with the empty vector (Figure 6J,K). These results indicate that overexpression of *lncRNA‐pys* enhances lignin biosynthesis and increases lignin content in transgenic pear callus.

In addition, one SNP genotype exhibited significant differences in expression levels (*p*‐value = 0.03, one‐way ANOVA). Notably, samples with the GG genotype showed higher expression levels compared to the AA genotype, suggesting that this variation may influence the transcriptional activity of *lncRNA‐pys*. These results support the idea that natural sequence variation within *lncRNA‐pys* can modulate its expression, which may in turn affect its regulatory role in lignin biosynthesis (Figure S26). In summary, these results suggest that selected lncRNAs have played a role in morphological and developmental changes during pear domestication and improvement.

To examine whether ectopic expression of pear *lncRNA‐pys* could affect lignin‐related traits in a heterologous system, we generated transgenic *Arabidopsis thaliana* Col‐0 plants overexpressing *lncRNA‐pys*. We identified nine hygromycin‐resistant T_1_‐generation transgenic plants on MS medium containing hygromycin (Figure S27). Homozygous T_3_‐generation plants derived from the three lines (OE‐1, OE‐3, OE‐9) were selected for experiments to assess their physiological and biochemical characteristics. At 20 days after transplantation, the number of leaves in wild‐type (WT) and *lncRNA‐pys*‐OE *Arabidopsis* plants was similar (Figure 7A,B). However, *lncRNA‐pys*‐OE plants exhibited delayed bolting compared to WT *Arabidopsis* (Figure 7A). By the 40^th^ day after transplantation, there were no significant differences between WT plants and *lncRNA‐pys*‐OE *Arabidopsis*. (Figure 7C,D). Notably, the lignin and cellulose contents were significantly higher in *lncRNA‐pys*‐OE *Arabidopsis* plants compared to WT plants (Figure 7E,F). qRT‐PCR analysis revealed that the expression of several lignin‐related genes, including *4CL2*, *CSE*, *CCOMT1*, *LAC1* and *POD2*, was significantly upregulated in transgenic lines compared with the WT plants (Figure S28). There were no observable differences in the morphology of interfascicular fiber and vessel cells, as indicated by toluidine blue O staining between WT plants and *lncRNA‐pys*‐OE *Arabidopsis* (Figure 7G,H). However, the xylem and interfascicular fiber in *lncRNA‐pys*‐OE *Arabidopsis* plants exhibited stronger spontaneous lignin autofluorescence than WT plants (Figure 7I,J). Furthermore, there was a more pronounced safranin O–fast green staining of lignin in the interfascicular fiber and xylem in *lncRNA‐pys*‐OE *Arabidopsis* plants compared to WT (Figure 7K,L). In addition, we observed the secondary cell walls (SCWs) of the interfascicular fiber and vessel cells using transmission electron microscopy (TEM). Compared to WT, the SCWs of the interfascicular fiber and vessel cells in *lncRNA‐pys*‐OE *A. thaliana* plants showed significant thickening (Figure 7M–P). These results indicate that ectopic expression of pear *lncRNA‐pys* in *Arabidopsis* is associated with increased lignin accumulation in the inflorescence stem.

**FIGURE 7:**
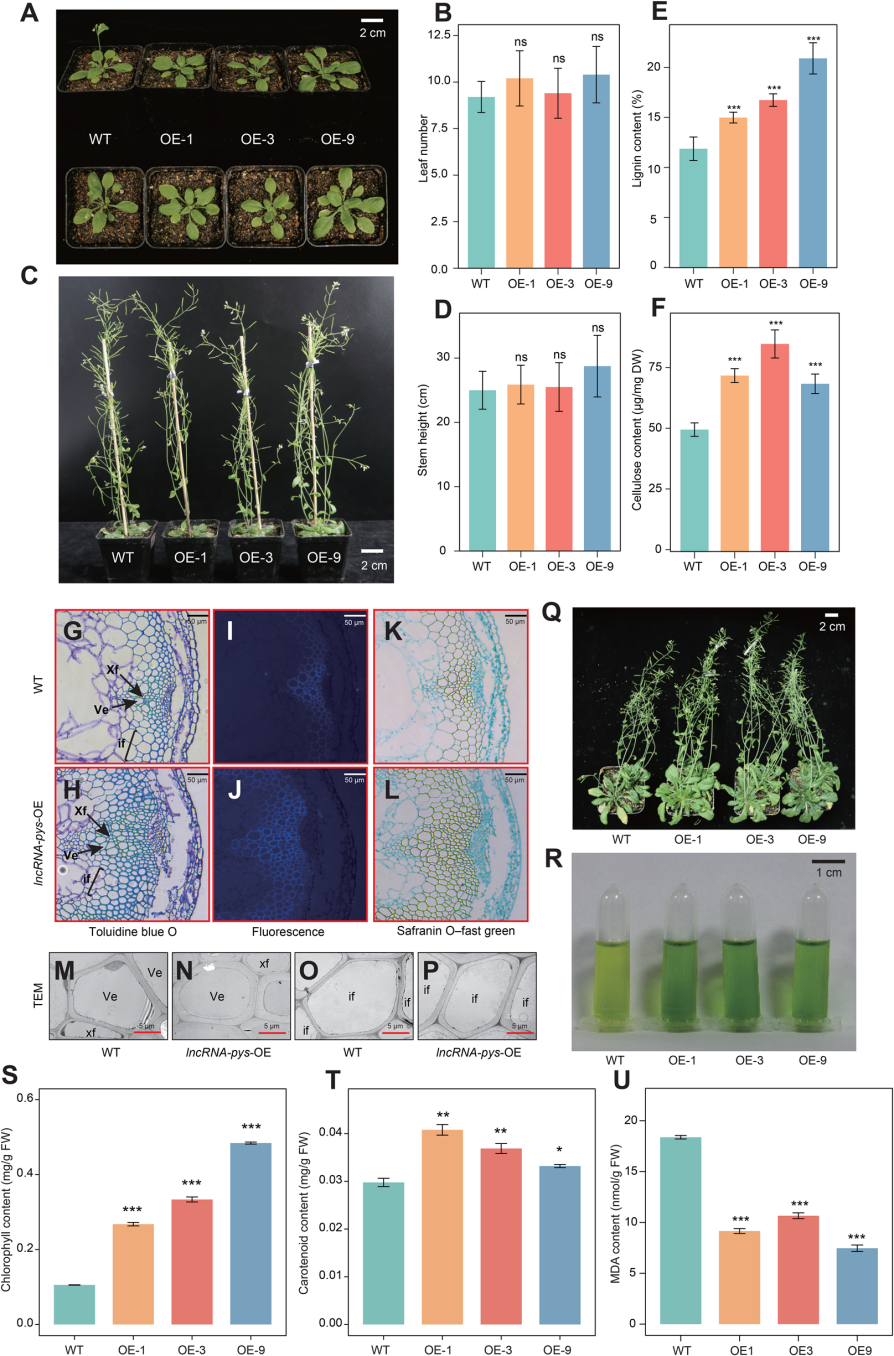
Functional validation of *lncRNA‐pys* in transgenic *Arabidopsis* plants. Image showing the leaves of representative WT *Arabidopsis* seedlings and those of three *lncRNA‐pys*‐OE *Arabidopsis* plants at 20 days old (A) and 40 days old (C) after transplantation. In sub part A, the top and bottom panels represent photographs of the same plant taken from different angles. The top panel shows the top view, while the bottom panel shows the side view. Quantification of WT and *lncRNA‐pys*‐OE *Arabidopsis* plants' morphology: leaf number at 20 days old (B) and stem height at 40 days old (D) after transplantation. (E) The lignin content (%) of *Arabidopsis* inflorescence stems in 8‐week‐old plants. (F) The cellulose content (%) of *Arabidopsis* inflorescence stems in 8‐week‐old plants. Data in (B), (D), (E) and (F) are presented as mean ± SD (*n* = 9 independent biological replicates). *p*‐values were tested using two‐sided unpaired Student's *t*‐tests. (*p < 0.05, **p < 0.01, ***p < 0.001, ns indicates no significant difference). Cross‐sections of inflorescence stems in WT (G) and *lncRNA‐pys*‐OE lines (H) were stained with toluidine blue O. Ve, vessel; Xf, xylary fiber; if, interfascicular fiber cell. Lignin auto‐fluorescence was detected under ultraviolet (UV) light in WT (I) and *lncRNA‐pys*‐OE lines (J). Cross‐sections of inflorescence stems in WT (K) and *lncRNA‐pys*‐OE lines (L) were stained with safranin O–fast green. Scale bars = 50 µm. (M–P) Transmission electron microscopy (TEM) of the cross‐section of cells. Scale bars = 5 µm. (Q) Phenotypes of wild‐type (WT) and transgenic (OE‐1, OE‐3, OE‐9) lines after 14 days of salt stress treatment. Scale bars = 2 cm. (R) Chlorophyll extraction from WT and transgenic lines after salt stress treatment. Scale bars = 1 cm. (S) Chlorophyll content in WT and transgenic lines after salt stress treatment. (T) Carotenoid content in WT and transgenic lines after salt stress treatment. (U) MDA content in WT and transgenic lines after salt stress treatment. Data in (S‐U) are presented as mean ± SD (*n* = 3 independent biological replicates). *p*‐values were tested using two‐sided unpaired Student's *t*‐tests (*p < 0.05, **p < 0.01, ***p < 0.001).

Given that lignin accumulation is associated with plant stress resistance, we examined the salt tolerance of T3 *Arabidopsis* overexpressing *lncRNA‐pys*. After 14 days of salt stress treatment, three *lncRNA‐pys*‐OE lines (OE‐1, OE‐3, OE‐9) showed better growth status compared to wild‐type (WT) plants (Figure 7Q). Additionally, the chlorophyll and carotenoid contents were significantly higher in the transgenic lines compared to WT, suggesting that overexpressing lines enhanced photosynthetic efficiency and salt tolerance (Figure 7R–T). The lower MDA content in the transgenic lines also indicated reduced membrane damage under salt stress conditions (Figure 7U). These results not only confirm the functional role of *lncRNA‐pys* in lignin biosynthesis, but also highlight its potential application in improving salt tolerance in pear.

## 3 Discussion

Non‐coding genomic regions have long remained enigmatic and were once regarded as “junk DNA” [39, 59]. However, recent research has uncovered their vital regulatory roles, such as the transcription of small RNA and lncRNA, which play a key role in gene expression regulation [60]. LncRNAs are emerging regulators pervasive throughout the genome [61]. Identifying lncRNAs accurately is challenging due to their low expression abundance [62]. In comparison to non‐strand RNA‐seq, ssRNA‐seq allows for the prediction of sense and antisense transcript structures, precise identification of overlapping transcription regions, and more accurate estimation of expression levels for both sense and antisense genes [63]. In our study, we utilized ssRNA‐seq to gather transcription direction information, which contributed to more precise estimation of lncRNA expression levels.

The characteristics of lncRNAs in pear are consistent with previous observations in model plants and mammals: (1) the full length of lncRNAs is, on average, shorter than that of PCGs; (2) lncRNAs tend to be predominantly mono‐exonic; (3) both exons and introns of lncRNAs are longer than those of PCGs; (4) lncRNAs typically exhibited lower expression levels, but greater expression diversity than PCGs; (5) lncRNAs display higher tissue‐specific expression compared to PCGs; (6) lncRNAs have a higher AU content than PCGs; (7) lncRNAs exhibit poor sequence conservation within the Rosaceae family; (8) lncRNAs are enriched in TEs when compared to PCGs. It is also worth noting that lncRNAs in pear contain a higher proportion of Long Terminal Repeat (LTR) type TEs. Interestingly, unlike previous findings in human and cotton where Long Interspersed Nuclear Element (LINE) elements were prominent in lncRNA transcripts [64, 65], we did not observe significant enrichment of LINE elements in pear lncRNAs. In fact, only a small fraction (0.5%) of lncRNA sequences in pear originated from LINE elements.

During the domestication of crops, not only was there a reduction in nucleotide diversity among cultivated varieties when compared to their relative wild counterparts [31, 32, 34], but there were also significant changes in gene expression patterns. For example, in maize, 3% of 18 242 genes examined exhibited significant differential expression when compared to its wild relative teosinte [66]. In sunflower (*Helianthus annuus*), Lai et al. reported that 5% of the total genes displayed differential expression between wild and weedy varieties [67]. In our study, we found 4522 (11%) and 5431 (13%) genes showed differential expression during the processes of pear domestication and improvement, respectively. Similarly, we observed that 1807 (15%) and 1828 (15%) lncRNAs exhibited significant differences in expression levels during pear domestication and improvement. These findings suggest that both genes and lncRNAs experienced a similar degree of selection pressure during the pear domestication and improvement processes. Notably, a significant trend we observed was that more lncRNAs were down‐regulated in both the domestication and improvement phases. This pattern resembles reports for rice [21].

Through the analysis of 41 resequenced pear genomes, we pinpointed selective sweep regions within the pear genome. We found that 857 lncRNAs were located within these regions during pear domestication, with 519 lncRNAs occurring during the improvement process. Furthermore, GO enrichment analysis of their target genes indicated that the selected lncRNAs in pear domestication and improvement were significantly associated with pear fruit size, sugar content, and stone cell content. In the case of rice, the target genes of down‐regulated DELs between wild and domesticated rice were linked to carbohydrate transport and reserve‐related functions, implying that the selection on lncRNAs led to increased starch content and grain weight [21]. Taken together, these findings provide robust evidence supporting the vital role of lncRNAs in the domestication of traits in crops.

The metabolome of landrace pears exhibited a 12.24% decrease in accumulation diversity compared to the wild population. In contrast, improved pears displayed a 36.05% increase in accumulation diversity compared to landrace pears, showing a distinct accumulation pattern during these two evolutionary processes. The recovery of diversity after domestication is a common phenomenon. For example, Hufford et al. reported the recovery of nucleotide diversity during the maize improvement process, likely because of introgression from wild relatives [30]. Li et al. also reported the recovery of expression diversity in genes during the pear improvement process [35]. In our study, we are the first to observe the recovery of accumulation diversity at the metabolome level during the pear improvement process. The increased diversity of metabolite accumulation in pears may facilitate adaptation to new environments and help meet the diverse demands of modern breeding programs. Notably, flavonoids, organic acids, saccharides and alcohols, and vitamin metabolites all experienced a recovery during the pear improvement process. Flavonoids are associated with plant resistance, while organic acids, saccharides and alcohols are linked to fruit taste, and vitamins play a vital role in immune function, vision, blood health, skin health and fetal development [68, 69, 70, 71, 72, 73]. Therefore, it is likely that diversifying selection during the improvement process was one of the important factors contributing to the recovery of accumulation diversity in the metabolome.

Stone cell content is an important domestication trait that substantially influences pear fruit quality [57, 74]. Pear stone cells are characterized by thick‐walled cells formed by parenchyma cells that deposit a substantial amount of lignin in their cell walls. Consequently, lignin synthesis plays a pivotal role in determining the accumulation of stone cells. In our current study, we observed that wild pears showed significantly higher concentrations of various intermediate products within the lignin biosynthetic pathway [75], including Caffeoyl‐p‐coumaroylquinic acid, Coniferyl alcohol, Chlorogenic Acid, 4‐Hydroxy‐3‐methoxycinnam aldehyde, p‐Coumaric acid, Coniferyl aldehyde, Sinapin aldehyde, and 3‐O‐p‐coumaroyl shikimic acid O‐hexoside. In contrast, landrace and improved pears showed relatively lower contents of these metabolites. Furthermore, our study revealed that five metabolites associated with lignin, including p‐Coumaric acid, p‐Coumaryl alcohol, Sinapin aldehyde, Coniferyl alcohol, and Coniferyl aldehyde, underwent reduced selection pressure during the pear domestication process. Additionally, seven Coumarins and Lignans metabolites experienced decreased selection pressure during both the pear domestication and improvement process. This study provides evidence for the negative selection of lignin‐related metabolites during domestication and improvement in pear. The changes in the levels of these metabolites may contribute to the decrease in stone cell content in pear fruit [76]. To gain further insights, we employed WGCNA to integrate the transcriptome and metabolome data from 41 pear accessions, constructing a co‐expression network of lncRNA‐mRNA to predict the regulatory roles of lncRNAs. As anticipated, we identified eight modules related to stone cell formation and further identified a group of lncRNAs within these modules that may influence the development of stone cells. For example, the *lncRNA‐pys* was identified as a regulator of *STONE* with the potential to influence stone cell formation [57]. Experimental verification analysis confirmed the significant role of *lncRNA‐pys* in lignin synthesis.

Although the precise molecular mechanism of *lncRNA‐pys* remains unresolved, our results suggest that it may function upstream of transcriptional activation of lignin‐related genes. The dual‐luciferase assays showed that *lncRNA‐pys* positively affected the promoter activities of several lignin‐associated genes, and the *Arabidopsis* overexpression lines displayed elevated expression of multiple lignin‐related structural genes, together supporting a positive regulatory role in lignin biosynthesis. Similar positive transcriptional regulation by plant lncRNAs has been reported previously. For example, the rice lncRNA *LAIR* enhances the promoter activities of neighboring *LRK* genes and is associated with active histone marks at the target locus [77], whereas the chrysanthemum NAT‐lncRNA *DglncTCP1* activates *DgTCP1* transcription by recruiting the histone modification factor *DgATX* and increasing H3K4me3 levels [25]. These studies suggest that plant lncRNAs may promote target‐gene transcription through diverse mechanisms, including transcriptional regulation and interactions with chromatin‐associated factors. Given that *lncRNA‐pys* enhances the promoter activities of several lignin‐associated genes, it may participate in the transcriptional regulation of lignin biosynthesis‐related genes. Whether *lncRNA‐pys* additionally functions through chromatin‐associated mechanisms, such as recruiting regulatory factors or influencing epigenetic modifications, remains to be further investigated.

## 4 Methods

### 4.1 Sample Materials

For the ssRNA‐seq analysis, we utilized fruit samples at the mature stage from a total of 41 sand pear accessions (*P. pyrifolia*) collected from the Wuhan Sand pear Germplasm Collection at the Hubei Academy of Agricultural Sciences, China. These 41 accessions consisted of 14 wild accessions (PYW1‐PYW14), 12 landrace accessions (PYL1‐PYL12), and 15 improved accessions (PYI1‐PYI15). To confirm the diversity of fruit‐related traits across the three groups, we analyzed 12 agronomic and fruit‐quality traits at the mature stage. As shown in Figure S1, wild pears exhibited significantly higher levels of stone cell content, lignin content, titratable acidity, and firmness, while improved pears showed lower levels of these traits but increased fruit size and sugar content. Recognizing the tissue‐specific nature of lncRNAs, and to comprehensively identify lncRNAs and investigate their expression changes during fruit development, we collected and performed ssRNA‐seq on samples from nine representative accessions at two additional key fruit development stages. These nine accessions were selected based on their genetic relationships and diversity, as supported by both phylogenetic analysis and nucleotide diversity (π) ranking (Figure S29 and Table S13), ensuring comprehensive representation of the genetic spectrum within the full panel. We selected 50 DAF and 90 DAF as the two key developmental stages based on previous anatomical and transcriptomic studies in pear fruit [35, 78]. The time point at 50 DAF corresponds to the peak period of stone cell and lignin accumulation, which plays a crucial role in determining fruit texture. The 90 DAF stage represents the phase when stone cell accumulation has completed, and fruit expansion becomes the dominant physiological process. Additionally, young leaves were also included in the analysis. All collected samples were immediately frozen in liquid nitrogen and stored at −80°C until RNA extraction.

### 4.2 RNA Extraction and Strand‐Specific Transcriptome Sequencing

To extract total RNA from the pulp of all pear samples, we employed the Plant Total RNA Isolation Kit Plus, as per the manufacturer's protocol (FOREGENE Co.), with three biological replicates for each sample. To monitor RNA quality and check for degradation and contamination, 1% agarose gels were used. RNA integrity was further assessed using the RNA Nano 6000 Assay Kit and the Bioanalyzer 2100 system by Agilent Technologies (Santa Clara, CA, USA). To remove ribosomal RNA, we utilized the Epicentre Ribo‐zero rRNA Removal Kit from Epicentre (Madison, WI, USA). Subsequently, strand‐specific RNA libraries were constructed with the NEBNext UltraTM Directional RNA Library Prep Kit for Illumina (NEB, Ipswich, MA, USA) using the rRNA‐depleted RNA samples according to the manufacturer's instructions. Sequencing was carried out on an Illumina HiSeq 4000 platform (Illumina, San Diego, CA, USA) to generate 150‐bp paired‐end reads.

### 4.3 Data Filtering and Mapping

To assess the quality of the reads in each sequence library, we employed the FastQC (v0.11.9) program (https://github.com/s‐andrews/FastQC). Adapter sequences, poly‐N stretches, and low‐quality reads were removed from the raw data using Trimmomatic (v0.39) with the following parameters: ILLUMINACLIP:TruSeq2‐PE.fa:2:40:15, LEADING:30, HEADCROP:10, TRAILING:30, SLIDINGWINDOW:4:15, AVGQUAL:30, and MINLEN:100 [79]. For sequence alignment, the reference genome of ‘Cuiguan’ pear (*P*. *pyrifolia*) was indexed using hisat2‐build. The alignment of the paired‐end clean reads was performed using HISAT2 (v2.2.1) with the –rna‐strandness RF parameter [41]. SAMtools (v1.2, using htslib 1.2.1) [80] was used to format conversion and sort the “bam” file, and (together with Picard) remove reads mapping to multiple positions, retaining only uniquely mapped reads for further analysis.

### 4.4 The Identification Pipeline of lncRNA

Transcript assembly was performed by independently de novo assembling the uniquely mapped reads from each sequencing library using the Cufflinks program (v2.2.1) with the –library‐type fr‐firststrand parameter [81]. The assembled transcripts from all 204 libraries were merged into a single GTF file using the Cuffmerge function in the Cufflinks package. This resulted in 190 683 transcripts originating from 63 155 loci.

To narrow down the selection to potential lncRNAs, we applied various filtering criteria, as these molecules are typically longer than 200 nucleotides. The Cuffcompare function from the Cufflinks package was used to examine the positional relationship between candidate lncRNAs and protein‐coding genes. Transcripts with one of the three class_codes (x, representing transcripts overlapping with reference exons on the opposite strand; i, indicating transcripts falling entirely within a reference intron; u, signifying intergenic transcripts of unknown function) were retained for further analysis, while 152 556 transcripts that overlapped with known features were removed. Furthermore, low‐expression lncRNAs were excluded based on the following criteria: single‐exon lncRNAs with an FPKM ≤ 2 and multi‐exon lncRNAs with an FPKM ≤ 0.5. Custom in‐house scripts used for transcript filtering are available at GitHub (https://github.com/Song820/lncRNA).

To evaluate the coding potential of transcripts, five different methods were employed, including PfamScan (http://ftp.ebi.ac.uk/pub/databases/Pfam/Tools/) [82], coding potential calculator (CPC) [83], predictor of long non‐coding RNAs and messenger RNAs based on an improved k‐mer scheme (PLEK) [84], Coding Non Coding Index (CNCI) [85] and Coding‐Potential Assessment Tool (CPAT) [86]. Pfam_scan.pl was used to identify known protein family domains in Pfam A database with the following parameters: −E 0.001 –domE 0.001. Pfam_scan.pl requires inputting the amino acid sequence, whereby we translated the nucleotide sequence of each transcript to the amino acid sequence using the EMBOSS software [87]. All the transcripts that yielded a Pfam hit in PfamScan were removed from further analysis. The other methods were applied with their respective default settings. In the identification process, transcripts predicted to be protein‐coding transcripts were removed from the set of lncRNAs. As a result, a total of 12 422 lncRNAs were identified within the pear genome. The genomic distribution of identified lncRNAs across pear chromosomes was visualized using Circos software (v0.69‐8).

### 4.5 Target Gene Prediction for lncRNAs

Because lncRNAs may affect gene expression, we tested for evidence of this effect. We calculated the expression correlation between lncRNAs and protein‐coding genes with in‐house scripts to identify a co‐expression pattern. If the protein‐coding genes were significantly co‐expressed with a given lncRNA, it indicated these genes may be regulated by this given lncRNA. We calculated the Pearson correlation of the expression between the protein‐coding genes and lncRNAs, and target gene threshold values were set as: |PCC| > 0.6, with a *p‐*value < 0.001. These co‐expressed protein‐coding genes were then used to perform GO enrichment analysis using the TBtools software, and significantly enriched GO terms were assigned to the corresponding lncRNAs [88].

### 4.6 Identification of Cis‐ and Trans‐Regulated lncRNA‐mRNA Pairs

To predict potential cis‐regulated target genes of lncRNAs, protein‐coding genes located within 10 kb upstream or downstream of each lncRNA locus were identified based on genomic coordinates. These neighboring genes were defined as putative cis target genes. To identify putative trans‐regulated lncRNA–mRNA pairs, expression correlations between lncRNAs and protein‐coding genes were calculated across all analyzed samples using the Pearson correlation coefficient (PCC). LncRNA–mRNA pairs with an absolute correlation coefficient |PCC| > 0.6 and *p*‐value < 0.001 were retained as putative trans‐regulated pairs.

### 4.7 Tissue Specificity Analysis

Tissue specificity scores were calculated for both lncRNAs and genes using an in‐house script (https://github.com/Song820/lncRNA). We used the expression matrix (FPKM values) derived from 68 samples to calculate tissue specificity scores. These included 41 mature fruit samples (14 wild, 12 landrace, and 15 improved accessions), 9 representative young fruit samples, 9 representative enlarged fruit samples, and 9 representative young leaf samples. 0 indicates housekeeping genes, and 1 indicates tissue‐restricted genes.

### 4.8 The Identification of TE‐Overlapping lncRNA

Transposable elements within the ‘Cuiguan’ pear genome were annotated using EDTA [89]. We analyzed 8 types of transposable elements, including DNA transposons, LINE, LTR, LTR/Copia, LTR/Gypsy, Helitron, MITE, and “Unknown”. The lncRNAs containing at least 10 nt transposable elements were defined as TE‐overlapping lncRNAs [90]. The “intersect” function of BEDtools was employed to calculate the length of overlap between lncRNAs and transposable elements [91].

### 4.9 TE Insertion Distribution Along Transcript (lncRNAs/PCGs) Regions

To investigate the distribution pattern of transposable element (TE) insertions relative to transcript structures, we analyzed TE overlap along transcript regions as follows. For each transcript (including PCGs and lncRNAs), we defined a region comprising the 2 kb upstream of the transcription start site (TSS), the full gene body, and the 2 kb downstream of the transcription termination site (TTS). This region was evenly divided into 80 bins (bin_1 to bin_80) to allow positional normalization across transcripts of different lengths. We then used the Bedtools (v2.30.0) coverage function to calculate the proportion of each bin that overlaps with annotated TE sequences. For each transcript type (e.g., NATs, lincRNAs, PCGs), the TE overlap ratio in each bin was averaged across all transcripts of this type. This resulted in a TE density profile along the normalized transcript structure, allowing us to compare TE insertion patterns among different gene categories.

### 4.10 Calculation of Sequence Conservation of Pear lncRNA Across Rosaceae Species

The genome sequences of six Rosaceae species were downloaded from the GDR database (https://www.rosaceae.org). The pear lncRNAs were aligned with the genomes of the following Rosaceae species*: Malus domestica* [92], *Rubus occidentalis* [93], *Prunus mume* [94], *Prunus persica* [95], *Fragaria vesca* [96], *Prunus avium* [97]. PCGs were used as a control. To perform the alignment, genome index databases for these six Rosaceae species were constructed using the “gmap_build” function in GMAP. Subsequently, alignment between pear lncRNAs and the six Rosaceae species was carried out using GMAP with the parameter “−k 15” [98]. For each lncRNA or PCG, only the best alignment hit was extracted. The conservation score for each transcript was determined by multiplying the sequence coverage by the identity. If an lncRNA or protein‐coding gene did not align with any of the selected species, its conservation score was defined as zero [99].

### 4.11 eTM Prediction

The miRNA sequences used in this study were obtained from our previous research [100]. The target genes of these miRNAs were predicted using the psRobot_tar software (v1.2), which identifies miRNA targets based on sequence complementarity [101]. For eTM identification, we used the eTMpy script available at GitHub (https://github.com/ztpub/eTMpy). The penalty score was set to ≤ 4 to define potential endogenous target mimics (eTMs). The miRNAs simultaneously targeting lncRNAs and protein‐coding genes were linked to construct the tripartite network.

### 4.12 Association Between DNA Methylation Variation and lncRNA Expression Variation

To determine if DNA methylation can explain expression variation, the whole‐genome bisulfite sequencing (WGBS) data of 41 pear fruits were obtained from a previous study [102]. Raw WGBS reads were first assessed for quality using FastQC (v0.11.9). Adapter sequences and low‐quality bases/reads were removed using Trimmomatic (v0.39). Clean paired‐end WGBS reads were then aligned to the ‘Cuiguan’ pear reference genome using Bismark. Duplicate reads were removed using deduplicate_bismark. Methylation calls were extracted from the deduplicated BAM files using bismark_methylation_extractor. For each lncRNA identified in our study, we examined whether accessions where a lncRNA was highly methylated showed significantly lower expression than accessions that were lowly methylated at this lncRNA locus (Mann–Whitney U test, *p* < 0.05). We calculated nine types of methylation level, including CG, CHG, and CHH methylation of body, and CG, CHG, and CHH methylation of upstream 2k, and CG, CHG, and CHH methylation of downstream 2k. The median of methylation level was used as threshold value to set “low” and “high” methylation (CG methylation: 0.6; CHG methylation: 0.53; CHH methylation: 0.39).

### 4.13 Differential Expression Analysis of lncRNAs

FPKM values were used to estimate the expression levels of lncRNAs/protein‐coding genes. Cufflinks was used to calculate the FPKM of each lncRNA and protein‐coding gene. We used R packages (“FactoMineR” and “factoextra”) to perform principal component analysis among the different groups [103]. We identified differentially expressed lncRNAs by applying the Cuffdiff function within Cufflinks [104]. The selection of significant differentially expressed lncRNAs was based on the following criteria: |log_2_(FoldChange)| ≥ 0.58; false discovery rate (FDR) ≤ 0.05.

### 4.14 Identification of Selective Sweep Regions During Pear Domestication and Improvement

We gathered young leaves from 41 sand pear accessions for genome resequencing. Quality control was performed using FastQC and Trimmomatic (parameter: ILLUMINACLIP:TruSeq3‐PE.fa:3:30:10, SLIDINGWINDOW:4:20, and MINLEN:50), resulting in clean reads that were subsequently aligned to the reference genome of the “Cuiguan” pear using the BWA program (parameter: ‐t 6 ‐k 32 ‐M) [41, 105]. For SNP calling, we employed the Genome Analysis Toolkit (GATK v4.1.4) following our established method [57, 106]. Low‐quality SNPs were filtered using VCFtools with the following parameters: “–maf 0.05; –max‐missing 0.9”. Furthermore, VCFtools was used to compute *F_ST_* and π (nucleotide diversity) with a window size of 10 kb and a window step size of 1 kb [107]. Candidate selective sweeps were identified in windows that ranked in the top 10% for both *F_ST_* (wild‐vs.‐landrace; landrace‐vs.‐improved) and the π ratio (π_wild_/π_landrace_; π_landrace_/π_improved_) [35]. LncRNAs and genes located within these selective sweep regions were categorized as selected lncRNAs and selected genes.

### 4.15 *Qst*‐*Fst* Analysis

We employed *Qst‐Fst* to identify which metabolites had undergone directed selection according to a previous study [108]. *Q_ST_* was an analog of *F_ST_* for phenotypic traits, defined using *Q_ST_* =σ_𝑏_2/(σ_w_2), while σ_𝑏_2 was between‐population variation, σ_w_2 was within‐population variation. The neutral *F_ST_* without bias was estimated using four‐fold synonymous transversion without missing values, and SNPs located in the selection region were removed. The distribution of σ_w_2 was obtained by multiplying observed σ_w_2 by a random number drawn from a χ^2^ distribution with a degree of freedom of one (number of population ‐ 1), then divided by five. The distribution of σ_𝑏_2 was given as σ_𝑏_2 = 2 *F_ST_* σ_w_2 / (1‐*F_ST_*). The neutral distribution of *Qst‐Fst* for each metabolite was calculated using 1000 replicates. The resulting *p‐*value was determined by the percentage of neutral *Qst‐F_ST_* distribution, which exhibited more extreme values than the observed *Qst‐Fst* value.

### 4.16 LC‐MS and Statistical Analysis of Metabolites

The untargeted metabolome was profiled at Wuhan Metware Biotechnology Co., Ltd. as previously described [109, 110, 111]. For the metabolomics analysis of ripe pear accessions, the pulp from all 41 samples was selected and analyzed using LC‐MS (liquid chromatography–mass spectrometry). The flesh from each sample underwent freeze‐drying and subsequent grinding to obtain a dry powder. Based on a previous study, 100 mg of dry powder was used for extraction and centrifugation, and the supernatant was filtered through a 0.22 µm filter. The filtered supernatant was then subjected to analysis on an ACQUITY Ultra Performance Liquid Chromatography (UPLC) system from Waters (Massachusetts, USA) coupled with an Agilent 6520 LC/MSD Ion Trap Mass Spectrometer (Agilent, Germany). Separation was performed on a 100 × 2.1 mm, 1.8 µm Waters ACQUITY UPLC HSS T3 column using a gradient of acetonitrile versus 0.1% formic acid in water. The gradient was run at a flow rate of 300 µL min and a temperature of 40°C at the following time points: 0 min, 5% acetonitrile; 2 min, 5% acetonitrile; 12 min, 95% acetonitrile; 15 min, 95% acetonitrile; 17 min, 5% acetonitrile; and 20 min, 5% acetonitrile. Quantification of the content of each metabolite was achieved by calculating peak areas and then standardizing the results using standard curves.

PLS‐DA was carried out using the “mixOmics” package in R, utilizing both the entire dataset of 2576 metabolomes and a subset of 517 annotated metabolomes (accessible at https://www.r‐project.org/). To assess the variation in all metabolites, the CV was computed with the help of R scripts. Differential expression analysis of metabolites was performed using MetaboAnalyst 5.0 [112]. This analysis involved comparing the wild‐vs.‐landrace and landrace‐vs.‐improved categories. The criteria for identifying differentially expressed metabolites were as follows: |log_2_(FoldChange)| ≥ 1, *p*‐value < 0.05.

### 4.17 Weighted Correlation Network Analysis and Construction of lncRNA‐Gene Co‐Expression Network

To generate co‐expression modules based on the differentially expressed lncRNAs and mRNAs in the comparisons of wild‐vs.‐landrace and landrace‐vs.‐improved, the WGCNA package in R was employed with default parameters, including a mergeCutHeight of 0.25 and a minModuleSize of 30 [113]. The soft threshold power was set to 5, resulting in a total of 52 co‐expression modules. The “grey” module represents the set of genes that were not grouped into any other module and was excluded from further analysis. Cytoscape was used to visualize the co‐expression networks between lncRNAs and genes [114].

### 4.18 Dual‐Luciferase Reporter Assays

To validate the interaction between lncRNA and target genes within the lncRNA‐mRNA co‐expression network, a dual‐luciferase (dual LUC) transient expression assay was conducted according to a previously established protocol [115]. The promoter sequences of the lignin‐related genes (*MYB24*, *STONE*, *4CL1*, *HCT1*, *LAC1* and *POD2*) were cloned into the pGreenII 0800‐LUC vector using *Kpn *I and *BamH *I restriction sites, creating the reporter constructs (*pro*::*LUC*). The effector (35S::*lncRNA*) was generated by inserting the lncRNA sequence at the N‐terminus of GFP under the control of the CaMV 35S promoter within the binary vector pCAMBIA1302 (p1302) (Table S12). All recombinant vectors used in this study were constructed using the ClonExpress Ultra One Step Cloning Kit V3 (Vazyme Biotech Co., Ltd) according to the manufacturer's instructions. The recombinant plasmids were then introduced into *Agrobacterium tumefaciens* GV3101. All recombinant plasmids were verified by Sanger sequencing and full‐plasmid next‐generation sequencing (NGS) performed by CwBio (Beijing, China) before downstream experiments. The bacteria were mixed and infiltrated into *Nicotiana benthamiana* leaves. After 40 h of infiltration, firefly luciferase (LUC) and renilla luciferase (REN) activities were measured using dual‐luciferase assay reagents from Promega (USA). LUC images were captured using a low‐light cooled CCD imaging apparatus (Tanon 5200 Multi) to assess the interactions and signaling within the dual‐LUC system.

### 4.19 Transient Transformation of *lncRNA‐pys* in Pear Fruit Flesh

To transiently overexpress *lncRNA‐pys*, the full‐length sequence of *lncRNA‐pys* was amplified using ApexHF HS DNA Polymerase CL (AG12204, Accurate Biotechnology Co., Ltd, ChangSha, China) and cloned into the overexpression vector. For virus‐induced gene silencing (VIGS), a specific fragment of *lncRNA‐pys* was amplified using the primers listed in Table S12 and inserted into the TRV2 vector. Transient overexpression and silencing assays in pear fruits were performed by *Agrobacterium tumefaciens*‐mediated infiltration. The fusion constructs and the empty control vector (p1300) were transformed into *Agrobacterium tumefaciens* strain GV3101 by the freeze–thaw method. For the transient transformation of pear fruit flesh, the cells were infiltrated into “Dangshansuli” fruit flesh at 35 DAF using needleless syringes. The infiltrated fruits were placed in the dark for 24 h and then incubated under a 16‐h light/8‐h dark photoperiod for 10 d. Six fruits were injected with each construct in each experiment, which was repeated three times independently. After 10 d, fruit sections were stained with phloroglucinol‐HCl (Wiesner's reagent) and photographed.

### 4.20 Genetic Transformation of Pear Callus

Transformation of pear callus was performed according to the method described in a previous study [116]. Pear callus was immersed for 15 min in MS liquid medium with a suspension of GV3101 cells (OD_600_ = 0.6) carrying either the *lncRNA‐pys* overexpression vector or the empty vector control. The infected pear callus was co‐cultured on solid MS medium for 48 h and then screened on solid MS subculture medium containing 20 mg/L hygromycin, 0.5 mg/L 6‐BA, and 1 mg/L 2,4‐D for at least 1 month in the dark at 24°C. Transgenic calli were subcultured every 15 d. Subsequently, the subcultured transgenic pear calli were moved to induction medium supplemented with different concentrations of EBR but lacking 2,4‐D and 6‐BA (Yamagishi et al. 2013) for 20 d and were then stained with phloroglucinol‐HCl (Wiesner's reagent).

### 4.21 *Arabidopsis* Transformation

To confirm the function of *lncRNA‐pys*, the floral dip method was employed to transform A*rabidopsis* Col‐0. *A*. tumefaciens GV3101 containing *lncRNA‐pys* overexpression constructs was used for the transformation. T_0_ seeds were screened for transformants by growing them on MS medium supplemented with 20 mg/L hygromycin. Subsequently, nine transgenic lines were successfully obtained after confirming the presence of *lncRNA‐pys* by PCR amplification using T_1_ plants. PCR amplification was performed using 2×Proofast Pro MasterMix (Dye) (P203‐3, ATG Biotechnology Co., Ltd, Nanjing, China) according to the manufacturer's instructions. PCR products were separated by agarose gel electrophoresis, and the sizes of DNA marker bands are indicated in Figure S27.

### 4.22 Measurement of Lignin Content and Microscopy

Inflorescence stems from both the WT and T3 *lncRNA‐pys*‐OE *Arabidopsis* plants were harvested, cut, and dried in an oven until a constant weight was achieved. Subsequently, 10 mg of the dried samples were extracted for lignin content analysis using a modified acetyl bromide method, as previously reported in our studies [57, 78]. For the basal portions of the primary inflorescence stem in both WT and *lncRNA‐pys*‐OE *A. thaliana* plants, they were immersed in a FAA (formalin–acetic acid–alcohol) fixative solution and kept at 4°C for 1 week. Following fixation, the sections were stained using toluidine blue O and safranin O–fast green. Lignified cell walls were observed using a Leica TCs SP2 spectral confocal microscope (Leica Microsystems, Germany). Lignin autofluorescence was visualized using ultraviolet light (excitation at 355/25) [58].

### 4.23 Salt Tolerance Assays of Transgenic Plants

To evaluate the salt tolerance of *lncRNA‐pys*‐OE *Arabidopsis* lines (OE‐1, OE‐3, OE‐9), *Arabidopsis* seeds of both transgenic and wild‐type lines were sown on solid MS medium. After 10 days of growth, seedlings were transplanted into pots filled with a 1:1 mixture of soil and vermiculite. Two seedlings were planted per pot, with six pots used for each transgenic and wild‐type line. After transplantation, the pots were covered with plastic film for three days to maintain humidity, followed by regular cultivation conditions. Plants were watered once every three days. After 25 days of growth, the following salt tolerance assays were conducted. After three days, a 100 mM NaCl solution was used for irrigation to induce salt stress. After 14 days of salt treatment, leaf samples were collected for physiological analyses. These included measurements of chlorophyll content, carotenoid content, and malondialdehyde (MDA) levels to assess photosynthetic efficiency and membrane damage under salt stress conditions. Chlorophyll and carotenoid concentrations were quantified using spectrophotometric methods. Malondialdehyde (MDA) content was measured using a commercial assay kit (Nanjing Jiancheng Bioengineering Institute, Nanjing, China) according to the manufacturer's instructions.

### 4.24 Statistical Analysis

Statistical analyses were performed using R (version 4.0.2) and Python (version 3.9.13). Unless otherwise indicated, quantitative data are presented as the mean ± standard deviation (SD) of independent biological replicates. The sample size (*n*) and the statistical test used for each analysis are specified in the corresponding figure legends. No data points were excluded unless explicitly stated. Comparisons between two independent groups were performed using two‐sided unpaired Student's *t*‐tests, whereas comparisons among three or more groups were performed using one‐way analysis of variance (ANOVA) to evaluate overall differences among groups. Duncan's multiple range test was used for multiple comparisons following ANOVA where indicated in the figure legends. The two‐sided Mann–Whitney U test was used for nonparametric comparisons between two independent groups as specified in the corresponding figure legends. Pearson's correlation coefficient was used to assess linear relationships between variables. Differences were considered statistically significant at *p* < 0.05.

## Author Contributions

J.W. designed and managed the project. B.S., J.F., and D.L. collected samples. B.S. performed data analyses. B.S. and P.H. performed the functional gene verification. X.L., J.L., X.Z., J.C., and L.Z. provided valuable suggestions. B.S. wrote the manuscript. J.W. revised the manuscript. All authors read and approved the final manuscript.

## Conflicts of Interest

The authors declare no conflicts of interest.

## Supporting information




**Supporting File 1**: advs77091‐sup‐0001‐TableS1‐S13.xlsx.

**Supporting File 2**: advs77091‐sup‐0002‐FigureS1‐S29.docx.

## Data Availability

The strand‐specific RNA sequencing (ssRNA‐seq) reads have been deposited into the NCBI Sequence Read Archive (SRA) (https://www.ncbi.nlm.nih.gov/sra/) under BioProject accession number PRJNA1041946; raw genome re‐sequencing reads have been deposited into the NCBI Sequence Read Archive (SRA) under BioProject accession number of PRJNA1041944. The lncRNA annotations, expression profiles, and DNA methylation datasets generated in this study are accessible through the Pear Genome Database (PGDB, http://pyrusgdb.sdau.edu.cn) and can be visualized using the integrated JBrowse genome browser (http://pyrusgdb.sdau.edu.cn/jbrowse/index.html?data=data/cuiguan_v1.0_lncRNA; http://pyrusgdb.sdau.edu.cn/jbrowse/index.html?data=data/cuiguan_v1.0_methylation). Custom scripts used in this study are available at GitHub: https://github.com/Song820/lncRNA.
