# Supplementary material for: The Landscape of Long Non‐Coding RNAs Provides Insights Into the Domestication and Improvement of Pear Fruit
Source: Adv Sci (Weinh). 2026 Aug 19;13(62):e77091. doi: 10.1002/advs.77091 (PMC13488485; doi:10.1002/advs.77091)
Supplement: Supplementary file 1 [file ADVS-13-e77091-s002.docx]

**Supplementary Figures 1-29**

Figure S1: The phenotype data of 41 pears. (A) 12 phenotypic traits of 41 pear accessions at the maturity stage were measured in our study, including fruit longitudinal diameter (cm), fruit transverse diameter (cm), single fruit weight (g), fruit-stem longitudinal diameter (cm), fruit-stem transverse diameter (cm), fruit-core longitudinal diameter (cm), fruit-core transverse diameter (cm), soluble solids content (%), stone cell (%), titratable acidity (%), fruit firmness (kg/cm2), vitamin C content (mg/100g). Statistical significance was assessed using two-sided unpaired Student’s t-tests. The sample sizes were n = 14, 12, and 15 for wild, landrace, and improved pears, respectively. Exact *P* values are shown above the box plots. Data are presented as box plots showing the median, interquartile range, and individual data points. (B) Correlation analysis of 12 phenotypic traits in wild, landrace and improvement populations, respectively. The color scale at the right represents Pearson correlation coefficients (PCC), with the corresponding values shown in each cell. Blue indicates positive correlation; dark red represents negative correlation.

Figure S2: The feature analysis of lncRNAs. (A) The mapping rate of wild, landrace and improved pear populations, respectively. The sample sizes were n = 14, 12, and 15 for wild, landrace, and improved pears, respectively. (B) Venn diagram representing the overlap number of lncRNA transcripts between pear lncRNAs and apple lncRNAs in the Plant long non-coding RNA database (PLncDB). (C) The classification of lncRNAs based on the relationship between the location of lncRNAs and protein-coding genes (PCGs).

Figure S3: The number of lncRNAs identified in different tissues and developmental stages of wild, landrace, and improved pear groups. The numbers of lncRNAs identified in leaf, young fruit, enlarged fruit, and mature fruit samples from wild, landrace, and improved pear groups are shown. Data are presented as box plots showing the median and interquartile range. The sample sizes varied among tissues and developmental stages. For mature fruit samples, the sample sizes were n = 15, 12, and 14 accessions for improved, landrace, and wild pear groups, respectively. For leaf, young fruit, and enlarged fruit samples, three accessions were analyzed for each pear group (n = 3).

Figure S4: The genomic distribution of lncRNAs and protein-coding genes (PCGs). (A) Chromosomal distribution of lncRNAs and PCGs. (B) Genome-wide relationships among transposable element abundance, PCG density, and lncRNA abundance. Pearson’s correlation coefficients (PCCs) and corresponding *P* values are shown above each panel.

Figure S5: Genome browser view showing the genomic locations of a protein-coding gene (EVM0019643.1), an antisense lncRNA (TCONS_00004201), and nearby transposable elements (TEs) on chromosome 1.

Figure S6: (A) The comparison of expression levels (log_2_(FPKM+1)) of nonTE_lncRNAs (lncRNAs without TE insertions) and TE_lncRNAs (lncRNAs with TE insertions). (B) The comparison of tissues specificity scores between nonTE_lncRNAs and TE_lncRNAs. (C) The comparison of transcript length of nonTE_lncRNAs and TE_lncRNAs. (D) The comparison of coefficient of variation (CV) for FPKM of nonTE_lncRNAs and TE_lncRNAs. For panels (A–D), the sample sizes were n = 3,195 and 9,227 for nonTE_lncRNAs and TE_lncRNAs, respectively. Statistical significance was assessed using two-sided unpaired Student’s t-tests (****P* < 0.001). (E) The comparison of CV among LTR-associated lncRNAs, Gypsy-associated lncRNAs, other TE-associated lncRNAs, and non-TE-associated lncRNAs. (F) The comparison of expression levels for FPKM among LTR-associated lncRNAs, Gypsy-associated lncRNAs, other TE-associated lncRNAs, and non-TE-associated lncRNAs. (G) The comparison of transcript length among LTR-associated lncRNAs, Gypsy-associated lncRNAs, other TE-associated lncRNAs, and non-TE-associated lncRNAs. (H) The comparison of tissues specificity scores by TEs among LTR-associated lncRNAs, Gypsy-associated lncRNAs, other TE-associated lncRNAs, and non-TE-associated lncRNAs. For panels (E–H), the sample sizes were n = 1,689, 1,363, 6,175, and 3,195 for LTR-associated lncRNAs, Gypsy-associated lncRNAs, other TE-associated lncRNAs, and non-TE-associated lncRNAs, respectively. Statistical significance was assessed using two-sided unpaired Student’s t-tests. **P* < 0.05, ***P* < 0.01, ****P* < 0.001 and *****P* < 0.0001.

Figure S7: Comparison of sequence conservation scores among different pear transcript types (protein-coding genes, incRNAs, lincRNAs, and NATs) across six Rosaceae species. Sequence conservation scores of protein-coding genes, incRNAs, lincRNAs, and NATs were compared across six Rosaceae species (*Malus domestica*, *Rubus occidentalis*, *Prunus mume*, *Prunus persica*, *Fragaria vesca*, and *Prunus avium*). Data are presented as box plots showing the median and interquartile range. The sample sizes were n = 42,622, 10,940, 564 and 918 for protein-coding genes, incRNAs, lincRNAs, and NATs, respectively. Statistical significance was assessed using one-way ANOVA followed by Duncan’s multiple range test. Different letters indicate significant differences among transcript types (*P* < 0.05).

Figure S8: Regulatory network of miRNA-lncRNA-mRNA interactions in pear.

Figure S9: DNA methylation level at upstream 2k (A) and downstream 2k (B) region of PCG, incRNA, lincRNA and NATs across 41 pear accessions. Data are presented as box plots showing the median and interquartile range. The sample sizes were n = 42,622, 10,940, 564 and 918 for protein-coding genes, incRNAs, lincRNAs, and NATs, respectively. *P* values were calculated using Mann-Whitney U test: **P* < 0.05, ***P* < 0.01, ****P* < 0.001, ns indicates no significant difference.

Figure S10: Gene expression explained by methylation level. (A) Summary of genes for which expression can be explained by methylation. The Venn diagrams show the overlap between loci for genes that were found to be defined by CG, CHG or CHH methylation levels. (B) An example of genes (*EVM0022826.1*) defined by CG, CHG or CHH methylation, showing RNA-seq signal, CG, CHG and CHH methylation levels in four accessions. Expression level as a function of CG, CHG and CHH methylation at upstream 2k (C) and downstream 2k (D) for the example gene (EVM0022826.1) across 41 pear accessions. The results of the Mann-Whitney U tests used for defining the explanatory power of CG, CHG and CHH methylation are shown (****P* < 0.001).

Figure S11: Chromosome distribution of DNA methylation-associated lncRNAs.

Figure S12: Comparison of tissue specificity scores between methylation-associated lncRNAs and non-methylation-associated lncRNAs. Data are presented as box plots showing the median and interquartile range. The sample sizes were n = 3,867 and n = 8,555 for methylation-associated lncRNAs and non-methylation-associated lncRNAs, respectively. Statistical significance was assessed using a two-sided unpaired Student’s t-test. The corresponding *P* value is shown above the box plots.

Figure S13: A principal variance component plots of expression levels across all and PCGs in wild, landrace and improved populations.

Figure S14: Volcano plot of differentially expressed lncRNAs and protein-coding genes between wild, landrace and improvement populations. (A) The differentially expressed lncRNAs between wild-vs-landrace, landrace-vs-improvement and wild-vs-improvement. (B) The differentially expressed protein-coding genes in comparisons between wild-vs-landrace, landrace-vs-improvement and wild-vs-improvement.

Figure S15: (A) GO enrichment analysis of target genes (4,192) of differentially expressed lncRNAs in wild-vs-landrace. (B) GO enrichment analysis of differentially expressed mRNAs in wild-vs-landrace. (C) GO enrichment analysis of target genes (7,890) of differentially expressed lncRNAs in landrace-vs-improved. (D) GO enrichment analysis of differentially expressed mRNAs in landrace-vs-improved. (E) Venn diagram showing the overlap between differentially expressed lncRNAs/PCGs between domestication and improvement processes, respectively.

Figure S16: Principal component analysis (PCA) of lncRNA-associated differentially expressed protein-coding genes during pear domestication and improvement. (A) PCA based on 450 protein-coding genes that are both differentially expressed and associated with differentially expressed lncRNAs in the wild vs landrace comparison. (B) PCA based on 1,078 protein-coding genes that are both differentially expressed and associated with differentially expressed lncRNAs in the landrace vs improved comparison.

Figure S17: Population genetic analysis of 41 pear accessions. (A) Principal component analysis of 41 pear accessions. (B) Phylogenetic analysis of 41 pear accessions based on whole genome SNP data obtained from resequencing data (Maximum-Likelihood tree, bootstraps = 1000). The 41 pear accessions were divided into three groups: wild, landrace and improved. The wild and landrace populations showed a degree of genetic mixing. (C) Population structure analysis of 41 pear accessions (K=2, 3, 4). (D) Fixation index (*F_ST_*) and nucleotide diversity (π) between wild, landrace and improved pear populations. (E) Linkage disequilibrium (LD) decay of wild, landrace and improved pear populations.

Figure S18. Distance-controlled analysis of selective signals in lncRNAs. LncRNAs were stratified into three categories according to their genomic distance to the nearest protein-coding gene (PCG): <5 kb, 5–20 kb, and >20 kb. Within each distance bin, selected lncRNAs showed significantly higher π ratio values than randomly sampled non-selected lncRNAs. Data are presented as box plots showing the median and interquartile range. Statistical significance was assessed using two-sided Mann-Whitney U tests. For domestication analysis, the sample sizes of selected and randomly sampled non-selected lncRNAs were n = 796 and 796 (<5 kb), n = 248 and 248 (5–20 kb), and n = 67 and 67 (>20 kb), respectively. For improvement analysis, the corresponding sample sizes were n = 409 and 409 (<5 kb), n = 115 and 115 (5–20 kb), and n = 31 and 31 (>20 kb), respectively. Asterisks indicate statistical significance (****P* < 0.001).

Figure S19: Metabolome divergence between wild, landrace and improvement pear populations. (A) PLS-DA of wild, landrace and improvement pears with 517 annotated metabolites. (B) The distribution of CV values in wild, landrace and improved pears. (C) The comparison of CV values between wild, landrace and improved pears across 14 metabolite classes. The sample sizes were n = 14, 12, and 15 for wild, landrace, and improved pears, respectively. (D) The heatmap of all differentially accumulated metabolites in wild-vs-landrace and landrace-vs-improved.

Figure S20: The construction of co-expression modules in WGCNA analysis. (A) Left, Analysis of the scale-free fit index for various soft-thresholding powers. The red line indicates where the correlation coefficient is 0.85, and the corresponding soft-thresholding power is 5. Right, Analysis of the mean connectivity for various soft-thresholding powers. (B) Dendrogram showing co-expression modules (clusters) identified by WGCNA during pear domestication and improvement processes. The major tree branches constitute 52 modules labeled with different colors.

Figure S21: Comparison of cellulose content among wild, landrace, and improved pear accessions. Data are presented as box plots showing the median and interquartile range (n = 14, 12, and 15 for wild, landrace, and improved groups, respectively). Statistical significance was assessed using two-sided unpaired Student’s t-tests for pairwise comparisons. Asterisks indicate significant differences (***P* < 0.01).

Figure S22: (A) GO enrichment analysis of mRNA in MEpink. (B) Putative transcriptional regulatory network of MEpink module.

Figure S23: Expression pattern of *lncRNA-pys* during fruit development of ‘Dangshansuli’. The expression level of *lncRNA-pys* was analyzed at five fruit developmental stages, including 11, 35, 60, 110, and 170 days after flowering (DAF). Data are presented as mean ± SD (n = 3 biological replicates).

Figure S24: Expression level of *lncRNA-pys* across three pear groups: wild, landrace, and improved accessions. Data are presented as box plots showing the median and interquartile range (n = 14, 12, and 15 for wild, landrace, and improved groups, respectively). Statistical significance was assessed using two-sided unpaired Student’s t-tests for pairwise comparisons. Asterisks indicate significant differences (**P* < 0.05, ns indicates no significant difference).

Figure S25: Correlation between the expression level of *lncRNA-pys* and three context (CG, CHG and CHH) methylation level at *STONE* gene promoter. Pearson’s correlation coefficients (PCCs) and corresponding *P* values are shown above each panel.

Figure S26: Association between sequence polymorphism and expression variation of *TCONS_00028382* (*lncRNA-pys*). (A) Genomic location and distribution of the SNP within the *lncRNA-pys* locus on chromosome 4, visualized using IGV. The polymorphic site is located at Chr4: 224,282 and is indicated by the red box. (B) The expression levels (FPKM) of *lncRNA-pys* in different SNP genotypes (0, AA, AG, GG). 0 represents missing genotype, AA and GG represent the two homozygous genotypes, and AG represents the heterozygous genotype at the polymorphic site. Data are presented as box plots showing the median, interquartile range, and individual data points (n = 3, 16, 3, and 19 for 0, AA, AG, and GG genotypes, respectively). Statistical significance was assessed using one-way ANOVA followed by Duncan’s multiple range test. The *P* value represents the overall difference among genotype groups, and different letters indicate significant differences among individual genotype groups (*P* < 0.05).

Figure S27: DNA amplification of *lncRNA-pys* in WT and *lncRNA-pys*-OE lines (OE-1 to OE-9). The sizes of the marker bands are indicated on the left.

Figure S28: qRT-PCR was used to the expression levels of genes related to lignin biosynthesis. Data are presented as mean ± SD (n = 3 independent biological replicates). Statistical significance was assessed using two-sided unpaired Student’s t-tests. Asterisks indicate significant differences (**P* < 0.05).

Figure S29: Phylogenetic analysis of 41 pear accessions based on whole genome SNP data obtained from resequencing data (Maximum-Likelihood tree, bootstraps = 1,000). The nine representative pear accessions were highlighted by red stars.
